# A meta-analysis and systematic review of creativity in schizophrenia: toward an ecological understanding integrating clinical and philosophical perspectives

**DOI:** 10.3389/fpsyg.2026.1658295

**Published:** 2026-03-04

**Authors:** Paola Pennisi, Federica Longo, Sara Alfia Nicotra, Mohammad Ali Salehinejad, Carmelo Mario Vicario, Alessandra Maria Falzone

**Affiliations:** 1 Department of Adult and Childhood Human Pathology “Gaetano Barresi”, Università degli Studi di Messina, Messina, Italy; 2 Department of Cognitive Sciences, Psychology, Education, and Cultural Studies, Università degli Studi di Messina, Messina, Italy; 3 Department of Child and Adolescent Psychiatry, Psychosomatics and Psychotherapy, Faculty of Medical, RWTH Aachen University, Aachen, Germany; 4 Cognitive Neuroscience Laboratory, Department of Cognitive Sciences, Psychology, Education, and Cultural Studies, Università degli Studi di Messina, Messina, Italy

**Keywords:** creativity, ecological assessment, mad-genius hypothesis, phenomenology, schizophrenia, originality, psychopathology

## Abstract

**Background:**

The presumed link between schizophrenia and creativity has long captured the collective imagination, but empirical data paint a more complex picture: while some patients produce extraordinary artistic works, quantitative studies consistently report lower creativity scores in individuals with schizophrenia compared to healthy controls. This contrasts with phenomenological accounts and clinical observations that highlight the expressive power of language and art in conveying the altered subjective experience of schizophrenia.

**Objective:**

This study aimed to update the existing evidence on creativity in schizophrenia through a systematic review and meta-analyses, and to assess whether a more fine-grained, ecologically valid approach might offer new insights.

**Methods:**

A systematic search of major databases yielded 4,043 studies after duplicate removal. Following PRISMA guidelines and strict inclusion criteria, 15 studies were included in the final qualitative synthesis and 13 in the quantitative meta-analyses. Creativity was analyzed both globally and across four subcomponents: elaboration, flexibility, fluency, and originality. Only tasks explicitly designed to elicit creative production were included.

**Results:**

All five meta-analyses showed statistically significant deficits in patients with schizophrenia compared to controls. The strongest effects emerged for overall creativity (*d* = –0.79), fluency (*d* = –0.83), and originality (*d* = –0.61). Moderator analyses revealed that age was the only significant variable: older patients showed larger deficits, particularly in fluency and flexibility. Other demographic and methodological factors did not account for variability in outcomes.

**Discussion:**

These findings confirm and extend prior work, suggesting that creativity is broadly impaired in schizophrenia. However, the consistent directionality of the results also raises critical questions about the ecological validity of standardized tests. Phenomenological and qualitative perspectives point to the importance of subjectivity and lived experience—dimensions often diminished by clinical treatments aimed at restoring shared reality. We call for the development of subjectivity-sensitive assessment tools capable of capturing this complexity.

**Conclusion:**

Creativity in schizophrenia remains a multidimensional phenomenon that cannot be fully understood through normative psychometric measures alone. Future research should adopt interdisciplinary approaches and develop novel tools for ecological assessment that are more sensitive to the creative potential of individuals with schizophrenia.

**Systematic review registration:**

https://www.crd.york.ac.uk/prospero/, CRD42024629254.

## 1 Introduction

According to the Diagnostic and Statistical Manual of Mental Disorders, Fifth Edition, Text Revision (DSM-5-TR), schizophrenia is a psychopathological condition characterized by delusions, hallucinations, disorganized speech (at least one of these must be present), as well as grossly disorganized or abnormal motor behavior, diminished affective expression, and abulia (American Psychiatric Association [APA], 2022). Within the clinical framework, creativity is understood as the ability to generate original and effective solutions to specific problems and in psychometric evaluations it is frequently assessed through four core dimensions of the Torrance Tests of Creative Thinking (TTCT) (Torrance, 1966): fluency (the number of responses produced per prompt), flexibility (the capacity to generate semantically diverse ideas), originality (the statistical rarity of proposed ideas) and elaboration (the degree of detail expressed in each response).

The idea of a relationship between madness and creative genius does not originate in modern psychopathology: rather, it has deep historical roots, with antecedents in antiquity, ideological elaboration during the Romantic era, and a contemporary reformulation within the frameworks of neuroscience and the psychology of creativity (Kyaga, 2015). However, while the collective imagination continues to romanticize the association between creativity and “madness” (Baudson, 2016), empirical data present a more nuanced story: individuals with psychotic disorders—particularly those diagnosed with schizophrenia—score significantly lower on standardized measure of creativity compared to healthy controls (cfr. Acar et al., 2018, for a meta-analysis on the topic).

Existing positions can be broadly grouped into three perspectives: (i) approaches that prioritize quantitative evidence and challenge the romantic association between creativity and madness (Dietrich, 2014); (ii) integrative approaches that attempt to reconcile this historical association with empirical findings (Abraham, 2014; Jung, 2014; Carson, 2011; Nettle and Clegg, 2006; Simonton, 2014); and (iii) phenomenological approaches that, by reflecting on the concept of creativity, challenge the explanatory adequacy of quantitative data (Sass, 2001).

The first (i) perspective accepts the empirical evidence and explains the persistence of the “mad genius” myth through statistical fallacies and cognitive biases. Dietrich for example (2014) argues that the perceived link between creativity and psychopathology is largely a product of base rate neglect, the availability heuristics, and confirmation bias. In his view, although some eminent creators may indeed have experienced mental disorders, the link between creativity and psychopathology is greatly overstated and lacks support from epidemiological evidence. He concludes that creativity is more plausibly linked to psychological wellbeing than to mental illness.

The second perspective (ii), while also accepting the empirical data, differs by proposing compensatory models to explain the observed patterns: these include the inverted U-shaped hypothesis between psychopathology and creativity (Abraham, 2014); the model of divergent evolutionary reasoning pressures (Jung, 2014); the shared vulnerability model (Carson, 2011); and the evolutionary hypothesis suggesting a reproductive advantage for subclinical schizotypal traits (Nettle and Clegg, 2006). Abraham (2014) posits a non-linear relationship between creativity and psychopathology, with creativity increasing under mild top-down control dysfunction and decreasing as dysfunction becomes more severe. This implies that creativity emerges from a balance between cognitive flexibility and executive control, placing schizophrenia at the lower end of the curve, where creative performance is most impaired. Jung (2014) conceptualizes creativity as an evolved cognitive mechanism for solving novel, non-recurrent problems through abductive reasoning, contrasting with the deductive processes used for familiar problems. In this framework, psychosis lies at one end of the cognitive spectrum, marked by excessive interconnectedness and self-referentiality, while extreme rule-based rigidity—often associated with autism spectrum disorders—lies at the other. The shared vulnerability model suggests that creativity and psychopathology are rooted in common cognitive traits such as disinhibition, atypical information processing, and neural hyperconnectivity (Carson, 2011). These traits can facilitate originality but also increase susceptibility to mental illness. In a similar vein, Nettle and Clegg (2006) propose that the same cognitive features that increase vulnerability to schizophrenia may, in milder forms, enhance creative potential. This supports the idea of an evolutionary trade-off, where moderate expression of schizotypal traits may be adaptive, while extreme forms lead to dysfunction. Simonton (2014), meanwhile, proposes that the relationship between creativity and psychopathology is not linear but variable: negative on average, yet positive among exceptional creators—thus explaining the discrepancy between psychometric studies and qualitative approaches.

Finally, a third perspective (iii), representing a more radical position, challenges the very foundations of how creativity is defined and measured. Louis Sass (Sass, 2001), for example, argues that, like “game,” creativity is not a single essence but what Wittgenstein (1953) called a *family resemblance concept*, that is, a category defined by a network of overlapping similarities, with no single feature common to all its members. For this reason, it depends on different historical, cultural, and stylistic contexts. This characteristic of creativity calls into question the claim of psychometric tests to measure a general and homogeneous capacity. Sass shows, for instance, how the twentieth-century avant-gardes (modernism and postmodernism) propose the idea that creation arises from detachment from the world, alienation, hyperreflexivity, fragmentation of the self, estrangement, and loss of natural self-evidence, all of which are strikingly resonant with the characteristics of schizophrenia (Spark et al., 2021; Parnas et al., 2005; Nelson and Raballo, 2015).

The present study aims to test the hypothesis that similarly divergent configurations might emerge in schizophrenia but can only be detected through a fine-grained analysis of creativity subcomponents. In contrast to the meta-analysis conducted by Acar et al. (2018), the present study is characterized by a twofold methodological intervention. First, it updates the aggregate data on creativity in individuals with schizophrenia, assessing whether the patterns identified in earlier studies persist in more recent literature. Second, it proposes a disaggregation of creativity into its core subcomponents—fluency, flexibility, originality, elaboration—but limits this analysis exclusively to studies in which these are assessed through tasks explicitly created to elicit creative production. In contrast to Acar et al. (2018), who included studies in which fluency or flexibility were measured in linguistic or attentional contexts unrelated to creativity per se, this study systematically excludes data derived from tasks that do not directly engage creative generation. This approach enables a more accurate distinction between general cognitive deficits and specific difficulties in creative production, thereby reducing the risk of overinterpreting linguistic measures as indicators of creativity. Our objective, therefore, is not only to update the existing literature but also to investigate whether individuals with schizophrenia exhibit a structurally distinct creative profile—one that could help explain, beyond anecdotal accounts, the production of radical creative works by some psychotic individuals. Should local peaks or anomalies emerge in specific creativity subcomponents, it might be hypothesized that schizophrenia does not simply impair creativity, but rather reorients it, as has already been observed in the context of autism (Pennisi et al., 2021).

### 2 Methods

The aim of this paper is to review and critically examine the current evidence on the relationship between schizophrenia and creativity. Adopting a quantitative approach, we used normative population averages as a reference point. We synthesized the results through a systematic review and conducted five meta-analyses. A systematic literature search was performed using Scopus, ScienceDirect, PubMed, and Web of Science. The search terms included “schizophrenia” and “creativity” as keywords in all fields (title, abstract, keywords, full text, and bibliography), with no restrictions on publication date.

**FIGURE 1:**
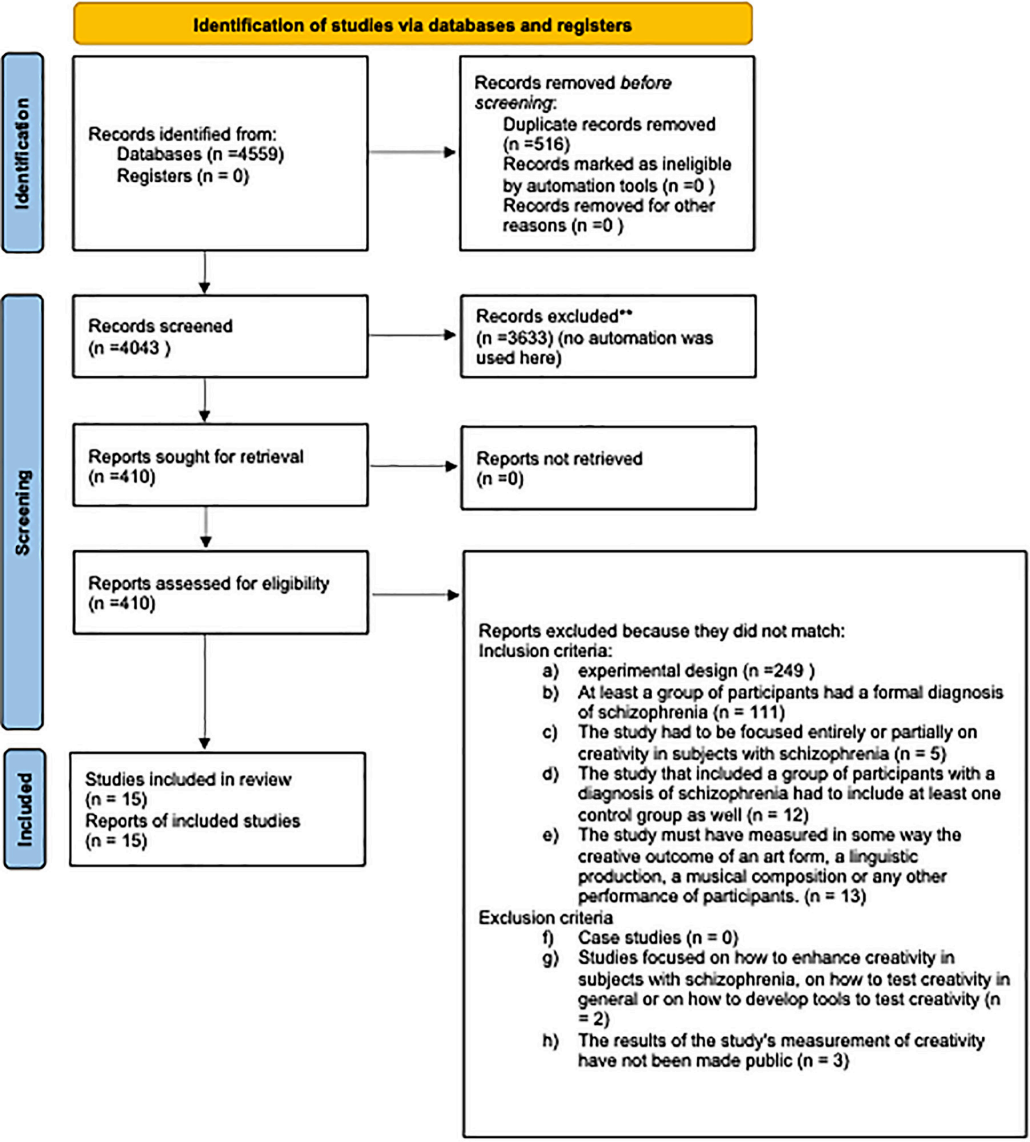
PRISMA 2020 flow diagram of included and excluded studies on creativity in schizophrenia. Source: Page et al. (2021).

The initial database search yielded 4,559 studies. After removing duplicates, 4,043 records remained. Included and excluded studies were collected following Preferred Reporting Items for Systematic Reviews and Meta Analyses (PRISMA) (Moher et al., 2009). The study selection process is summarized in the PRISMA flow diagram (Figure 1). This review was prospectively registered in the PROSPERO database (the registration ID is: CRD42024629254).

### 2.1 Screening and eligibility criteria

The initial screening of titles and abstracts was independently conducted by the second and third authors. The primary objective at this stage was to exclude studies not directly centered on creativity in schizophrenia. Inter-rater reliability for study inclusion was assessed using Cohen’s Kappa, yielding a value of 0.854, indicating near-perfect agreement. The observed agreement was 97.0%, with an expected agreement of 79.4%.

Following a collective discussion among the first, second, and third authors, a consensus was reached to exclude the 122 studies that were not mutually agreed upon by the initial reviewers. As a result, the first screening led to the exclusion of 3,633 records. After the screening, we assessed 410 results for eligibility.

The following is the full list of inclusion and exclusion criteria for assessing eligibility.

Inclusion criteria:

a. Experimental design;
b. At least a group of participants had a formal diagnosis of schizophrenia (according to ICD or DSM in any version);
c. The study had to be focused entirely or partially on creativity in subjects with schizophrenia;
d. The study that included a group of participants with a diagnosis of schizophrenia had to include at least one control group as well;
e. The study must have measured in some way the creative outcome of an art form, a linguistic production, a musical composition, or any other performance of participants.

Exclusion criteria:

f. Case studies ( ≤ 2 participants);
g. Studies focused on how to enhance creativity in subjects with schizophrenia, on how to test creativity in general, or on how to develop tools to test creativity;
h. The results of the study’s measurement of creativity have not been made public.

### 2.2 Final sample

During the selection of the final sample from the 410 studies included in the second screening phase, inter-rater agreement between the second and third authors was nearly perfect, with a Cohen’s Kappa of 0.926 (observed agreement = 99.5%, expected agreement = 93.4%). Two disagreements arose, each concerning a different study, and were resolved through a consensus discussion involving the first, second, and third authors, resulting in a final sample of 15 studies.

During the eligibility assessment, we excluded 390 results because they did not meet the inclusion criteria (*a* = 249; *b* = 111; *c* = 5; *d* = 12; *e* = 13) and 5 results because they met our exclusion criteria (*f* = 0; *g* = 2; *h* = 3). The eligibility assessment led to the exclusion of a total of 395 results. Our final sample consisted of 15 articles.

### 2.3 Quality assessment

Almost all the included studies were observational and non-randomized. Although the study of Shimkunas and Murray (1974) employed a quasi-experimental design, none of the studies involved random allocation or interventional manipulation. Given the nature of these studies-primarily cross-sectional comparisons between predefined clinical and non-clinical groups risk of bias was assessed using the AXIS tool (Appraisal tool for Cross-Sectional Studies), developed by Downes et al. (2016). The AXIS tool is specifically designed to evaluate methodological quality and risk of bias in cross-sectional studies, addressing key domains such as study design, sample selection, measurement validity, and transparency of reporting. Its structured approach allows for the consistent and context-appropriate appraisal of studies that assess psychological, behavioral, or cognitive outcomes without experimental intervention.

A score of 1 was assigned to all “Yes” responses, except for items 13 and 19, for which a score of 1 was assigned to “No” responses, as in these cases “No” indicates higher study quality. Papers were classified as low (0–7), moderate (8–14), or high (15–20) quality, based on classifications used in previous literature (Moor and Anderson, 2019; Musetti et al., 2021; Chellew et al., 2022).

Based on our criteria, all studies included in the sample were rated as “high quality”. The only exception was the paper by Folley and Park (2005), which received a score of 14, placing it at the upper threshold of the “moderate” quality category. However, it is important to note that, while the AXIS tool typically ranges from 0 to 20, in all studies analyzed, item 14 (“Were non-responders described?”) was consistently marked as “not appropriate,” since no participants in any of the studies had failed to respond to the relevant measures. As a result, the maximum achievable score across the sample was 19. Therefore, these two studies can be considered borderline between the “moderate” and “high” quality categories. Detailed results of the quality assessment are presented in Table 1.

**TABLE 1:** Quality assessment of selected studies (Appraisal tool for Cross-Sectional Studies).

Study	Introduction	Methods										Results					Discussion		Other		Results	
	1	2	3	4	5	6	7	8	9	10	11	12	13	14	15	16	17	18	19	20	Tot	Classification
Abraham et al. (2007)	1	1	0	1	0	1	0	1	1	1	1	1	1	NA	1	1	1	0	1	1	15	High quality
Del Missier et al. (2022)	1	1	0	1	1	1	0	1	1	1	1	1	1	NA	1	1	1	1	1	1	17	High quality
Folley and Park (2005)	1	1	0	1	0	0	0	1	1	1	1	1	1	NA	1	1	1	0	1	1	14	Moderate quality
Jaracz et al. (2012)	1	1	0	1	0	0	0	1	1	1	1	1	1	NA	1	1	1	1	1	1	15	High quality
Keefe and Magaro (1980)	1	1	0	1	0	1	0	1	1	1	1	1	1	NA	1	1	1	1	1	0	15	High quality
Kucwaj et al. (2023)	1	1	0	1	0	1	0	1	1	1	1	0	1	NA	1	1	1	1	1	1	15	High quality
Mavrogiorgou et al. (2021)	1	1	0	1	0	0	0	1	1	1	1	1	1	NA	1	1	1	1	1	1	15	High quality
Michalica and Hunt (2013)	1	1	0	1	0	0	0	1	1	1	1	1	1	NA	1	1	1	1	1	1	15	High quality
Rodrigue and Perkins (2012)	1	1	0	1	0	0	0	1	1	1	1	1	1	NA	1	1	1	1	1	1	15	High quality
Rubinstein (2008)	1	1	0	1	1	1	0	1	1	1	1	1	1	NA	1	1	1	1	0	1	16	High quality
Salesse et al. (2021)	1	1	0	1	0	1	0	1	1	1	1	1	1	NA	1	1	1	1	1	1	16	High quality
Sampedro et al. (2019)	1	1	0	1	1	1	0	1	1	1	1	1	1	NA	1	1	1	1	1	1	17	High quality
Shimkunas and Murray (1974)	1	1	0	1	1	0	1	1	1	1	1	1	1	NA	1	1	1	0	1	0	15	High quality
Son et al. (2015)	1	1	0	1	0	1	0	1	1	1	1	1	1	NA	1	1	1	1	1	1	16	High quality
Wang et al. (2017)	1	1	0	1	0	1	0	1	1	1	1	1	1	NA	1	1	1	1	1	1	16	High quality

### 2.4 Outcomes

For the meta-analyses, data on creativity were extracted from 11 studies (Abraham et al., 2007; Del Missier et al., 2022; Folley and Park, 2005; Jaracz et al., 2012; Keefe and Magaro, 1980; Mavrogiorgou et al., 2021; Michalica and Hunt, 2013; Rodrigue and Perkins, 2012; Sampedro et al., 2019; Wang et al., 2017), data on elaboration from 5 studies (Abraham et al., 2007; Mavrogiorgou et al., 2021; Sampedro et al., 2019; Wang et al., 2017), data on flexibility from 7 studies (Del Missier et al., 2022; Folley and Park, 2005; Jaracz et al., 2012; Kucwaj et al., 2023; Mavrogiorgou et al., 2021; Wang et al., 2017), data on fluency from 8 studies (Abraham et al., 2007; Del Missier et al., 2022; Jaracz et al., 2012; Keefe and Magaro, 1980; Mavrogiorgou et al., 2021; Salesse et al., 2021; Sampedro et al., 2019; Son et al., 2015; Wang et al., 2017), and data on originality from 5 studies (Abraham et al., 2007; Del Missier et al., 2022; Mavrogiorgou et al., 2021; Sampedro et al., 2019; Wang et al., 2017). Following the methodological guidance by Borenstein et al. (2021), when a primary study reported two distinct scores for the same outcome, we computed a combined value by averaging the two means and estimating the pooled standard deviation (normally assuming *r* = 0.05). Detailed information on how the scores for each individual outcome were obtained in ambiguous cases is fully reported in Supplementary material S2. Details on how we coded all the variables of outcomes are reported in Supplementary Tables 1–5.

### 2.5 Statistical analyses

In line with meta-analytic recommendations, we (a) collected data from each single study; (b) calculated standardized mean difference effect sizes for each comparison (Cohen’s *d*) (Cohen, 2013); (c) determined the overall effect sizes for each comparison with a random effect model; (d) identified potential moderator variables; (e) measured heterogeneity through I^2^ (Higgins et al., 2024; Rosenthal, 1979); and (f) used the Egger’s linear regression method (Egger et al., 1997) and the Duval and Tweedie’s trim and fill procedure (Duval and Tweedie, 2000) in order to evaluate publication bias.

We conducted a meta-analysis focusing on five outcomes: creativity, elaboration, flexibility, fluency, and originality. In all five cases, the null hypotheses were structured such that a lower performance in the experimental group relative to the control group would result in a negative overall effect size. We also examined the following moderators: year of publication, mean age of the experimental group, mean age of the control group, age difference between the experimental and control groups, proportion of male participants in the sample, mean years of education in the patient group, and the difference in educational attainment between patients and controls.

To evaluate the significance of the results, we used Cohen’s (1988) guidelines: where *d* = 0.00 is a null effect; *d* = 0.20 is a small effect; *d* = 0.50 is a medium effect; *d* = 0.80 is a big effect. In this framework, a positive d indicates that the group with schizophrenia scored higher than the control group, while a negative d value indicates that the group with schizophrenia scored lower than the control group.

To interpret heterogeneity, we used Higgins et al. (2024) parameters: *I*^2^ = 0–24 as negligible heterogeneity; *I*^2^ = 25–49 as low; *I*^2^ = 50–74 as moderate; and *I*^2^ = 75–100 as high heterogeneity. For testing of the first hypothesis, we analysed a sample of 11 studies (*k* = 11); in this case, the trim and fill analysis is considered more reliable than the fail-safe N. Conversely, for all other hypotheses where *k* < 10, the fail-safe N is likely more dependable than the trim and fill approach, which relies on a funnel plot analysis.

## 3 Results

Table 2 presents the PICOS framework, including detailed information on the study populations, intervention (*task* in this case), comparator, outcomes, study design. Summary results for the overall effect sizes of all meta-analyses are presented in Table 3, while heterogeneity statistics are detailed in Table 4. The entire sample for the systematic included 15 works (Abraham et al., 2007; Del Missier et al., 2022; Folley and Park, 2005; Jaracz et al., 2012; Keefe and Magaro, 1980; Kucwaj et al., 2023; Mavrogiorgou et al., 2021; Michalica and Hunt, 2013; Rodrigue and Perkins, 2012; Rubinstein, 2008; Salesse et al., 2021; Sampedro et al., 2019; Shimkunas and Murray, 1974; Son et al., 2015; Wang et al., 2017). Two studies were excluded from the meta-analysis. Rubinstein (2008) was excluded because it was the only study that included only psychiatric patients as controls, without a healthy control group. As only one other study (Mavrogiorgou et al., 2021) used a psychiatric control group, the available data were insufficient for comparison between individuals with schizophrenia and other psychiatric patients. Shimkunas and Murray (1974) was also excluded due to scoring methods that were inconsistent with our definition of creativity.

**TABLE 2:** PICOS framework of the reviewed studies.

Study	Population	Task	Comparator	Outcomes	Study design
Abraham et al. (2007)	28 patients with schizophrenia Age: 43.07 (9.91) Sex: 23 males and 5 females; male proportion: 0.821 IQ: 108.11 (92–123) All participants were medicated at the time of testing with clozapine	Ward animal task (Ward, 1994) to assess conceptual expansion Creative imagery (Abraham et al., 2007; Finke, 1990) Constraints of examples. This task asks participants to imagine new ideas for toys Alternate uses task (based on Wallach and Kogan, 1965) Convergent problem solving	18 healthy controls Age: 39.11 (12.54) Sex: 14 males and 4 females IQ: 111.44 (92–122)	Creativity Elaboration Fluency Originality	Comparative cross-sectional case-control design
Del Missier et al. (2022)	17 participants with schizophrenia Age: 51.94 (9.90) Sex: 11 males and 6 females; male proportion: 0.647 All participants were on antipsychotic medication at the time of testing; 12 also took anxiolytics, and 7 were on other medications.	Alternative Uses Test to assess divergent thinking (Guilford et al., 1978) Remote Association Test to assess convergent thinking (Mednick, 1962)	17 healthy controls Age: 51.76 (11.32) Sex: 11 males, 6 females	Creativity Flexibility Fluency Originality	Comparative cross-sectional case-control design
Folley and Park (2005)	17 subjects with schizophrenia Age: 39.5 (2.6) Sex: 12 males and 5 females; male proportion: 0.706 Handedness*: all right-handed Years of education*: 13.0 (0.5) IQ* All participants were receiving antipsychotic medication at the time of testing with antipsychotic drugs	Remote Associates Test (Mednick, 1962) A novel divergent thinking task (subjects had to generate uses for real objects)	17 Subjects with schizotypal personality Age: 22.8 (1.8) Sex: 9 males and 8 females Handedness*: all right-handed Years of education*: 13.9 (0.3) IQ* 17 Healthy controls Age: 35.2 (3.1) Sex: 9 males and 8 females Handedness*: all right-handed Years of education*: 12.9 (0.3) IQ*	Creativity Flexibility	Comparative cross-sectional multi-group design
Jaracz et al. (2012)	43 participants with schizophrenia Age: 36 (10; 21–60) Sex: 22 males and 21 females; male proportion: 0.512 Years of education: 12 (2; 8–18) All the patients were on antipsychotic medication at the time of testing. Eleven patients were treated with first generation neuroleptics (haloperidol, perphenazine), others received atypical antipsychotics (clozapine, olanzapine, quetiapine, and risperidone).	Barron-Welsh Art Scale (BWAS, Barron, 1963; Barron and Welsh, 1952) to assess creativity Berlin Intelligence Structure Test (BIS, Jäger et al., 1997) to assess creativity	45 healthy controls Age: 35 (12; 18–60) Sex: 17 males and 28 females Years of education: 15 (4; 7-25)	Creativity Flexibility Fluency	Comparative cross-sectional case-control design
Keefe and Magaro (1980)	10 participants with paranoid schizophrenia Age: 23.80 (3.60) Years of education: 11.50 (1.06) 10 participants with non-paranoid schizophrenia Age: 23.80 (4.02) Years of education: 12.00 (2.12) All the patients were on antipsychotic medication at the time of testing.	Alternate Uses Test. Two scoring indexes were used: the total number of responses by subject and the sum of the assessed creativity on each response (from zero to three) Revised Art Scale of the Barron-Welsh Figure Preference Test (Barron and Welsh, 1952)	10 non-psychotic psychiatric patients Age: 29.20 (10.23) Years of education: 11.70 (2.40) 10 healthy controls Age: 33.40 (8.91) Years of education: 13.80 (2.41)	Creativity Fluency	Comparative cross-sectional multi-group design
Kucwaj et al. (2023)	62 participants with schizophrenia Age: 29.35 (5.24; 19–38) Sex: 43 males and 18 females; male proportion: 0.694 Years of education: 8 (5.24; 4–14) All patients were treated with second-generation antipsychotics	Cattell Culture Fair Intelligence Test Six Remote Associates Test Insight problem to solve through the manipulation of objects	62 healthy controls Age: 27.74 (5.71; 18–38) Sex: 19 men and 43 women Years of education: 12 (4.85; 4–14)	Flexibility	Comparative cross-sectional case-control design
Mavrogiorgou et al. (2021)	10 participants with schizophrenia Age: 40.4 (13.5; 18–67) All patients were medicated, generally with second generation antipsychotics but sometimes combined with a benzodiazepine.	Torrance Tests of Creative Thinking (TTCT, Torrance, 1966)	15 participants with affective disorder Age: 45.4 (20.5; 18–75) 25 healthy controls Age: 46.1 (15.4; 20–68) Years of education: 9.28 (3.14; 4–12)	Creativity Elaboration Flexibility Fluency Originality	Comparative cross-sectional multi-group design
Michalica and Hunt (2013)	10 participants with schizophrenia Age: 37.7 (8.75; 25–60) Sex: 7 males and 3 females; male proportion: 0.7 No information is provided regarding any pharmacological treatment of the participants with schizophrenia.	Barron-Welsh Art Scales (Barron and Welsh, 1952) to assess creativity Self-rating of creativity on a five-point Likert scale	31 healthy artists Age: 43.1 (14.25; 21–78) Sex: 14 males and 17 females 31 healthy participants matched for age and sex with artists Age: 39.4 (8.75; 23–76) Sex: 12 males and 19 females 102 healthy controls used to examine the same variables in a normal population Age: 19.8 (2.75; 17–28) Sex: 25 males and 77 females	Creativity	Comparative cross-sectional multi-group design
Rodrigue and Perkins, 2012	22 participants with schizophrenia Age: 43.7 Sex: 15 males and 7 females; male proportion: 0.682 Years of education: 11.5 All participants were medicated at the time of testing	Abbreviated Torrance Test (Goff and Torrance, 2002) to assess divergent thinking	30 healthy control participants Age: 19.8 Sex: 10 males and 20 females 30 psychometrically determined schizotypal healthy participants Age: 19.9 Sex: 10 males and 20 females	Creativity	Comparative cross-sectional multi-group design
Rubinstein (2008)	88 participants with schizophrenia Age: 29.00 (3.13) range from 25 to 40 Sex: 84 males and 86 females Religion: 62 Jewish, 49 Christian, 59 Muslim*	Tel-Aviv Creativity Test	57 participants suffering from anxiety, depression or both 46 Personality disordered participants (clusters A and B)	Excluded from the meta-analyses due to the lack of healthy control group	Comparative cross-sectional multi-group design
Salesse et al. (2021)	30 participants with schizophrenia Sex: 28 males and 2 females; male proportion: 0.933 Age: 33.8 (10.4; 18–58) Years of education: 14.2 (3.3; 3–6) All patients received anti-psychotic medication	Originality in performing motor actions was assessed in three conditions (within individual, inter-individual and social levels) during an imitation game.	28 healthy control participants Sex: 25 males and 3 females Age: 31.6 (5.7; 24–49) Years of education: 14.2 (2.1; 3–6)	Fluency	Comparative cross-sectional case-control design
Sampedro et al. (2019)	45 participants with schizophrenia Age: 40.4 (13.5; 18–67) Years of education: 10.56 All patients with schizophrenia were receiving antipsychotic treatment.	Picture Completion subtest from the Figural Form of the TTCT (Torrance, 1966) Unusual Uses subtests from the Verbal Form of the TTCT (Torrance, 1966)	45 healthy controls Age: 38.91 (14.67) Sex: 15 males and 30 females Years of education: 14.67 (3.58)	Creativity Elaboration Fluency Originality	Comparative cross-sectional case-control design
Shimkunas and Murray (1974)	24 participants with schizophrenia Age: 37.55 (10.99) Sex: 11 males and 13 females)	Guilford’s Alternate Uses Test (Guilford, 1954) with a different scoring. Authors organized responses in three categories: *conventional*, *imaginative* and *clang or illogical*	24 non-psychotic patients Age: 35.95 (12.01) Sex: 10 males and 14 females	Excluded due to inconsistency between the scoring method and our definition of creativity	Comparative cross-sectional case-control design
Son et al. (2015)	43 subjects with schizophrenia Age*: 37.37 (8.66) Sex*: 22 males and 21 females; male proportion: 0.512 Handedness*: 73.49 (46.70) Premorbid IQ*: 102.88 (9.80) All patients were receiving antipsychotic medication (typical [*n* = 3], atypical [*n* = 31], typical and atypical [n = 9]).	Idea, design and verbal (semantic and phonological) fluency tests	36 Healthy Controls Age*: 33.86 (8.66) Sex*: 24 males and 12 females Handedness*: 81.94 (35.52) IQ*: 106.47 (7.62)	Fluency	Comparative cross-sectional case-control design
Wang et al. (2017)	43 participants with schizophrenia Age: 30.23 (6.12; 18–40) Sex: 19 males and 24 females; male proportion: 0.442 Years of education: 14.67 (2.39) All patients were taking atypical antipsychotic drugs	Alternative use task and figure completion task adapted from the TTCT to assess divergent thinking (Torrance, 1966) Insight problem solving tasks to assess convergent thinking Tasks that require to generate visual-spatial and verbal products adapted from Domino (1980) and Howard-Jones et al. (2005) to assess together divergent and convergent thinking	39 psychometrically determined low-schizotypal healthy participants Age: 20.69 (1.47) Sex: 5 males and 34 females Years of education: 14.13 (1.26) 35 psychometrically determined high-schizotypal healthy participants Age: 20.31 (1.35) Sex: 5 males and 30 females Years of education: 13.89 (1.35)	Creativity Elaboration Flexibility Fluency Originality	Comparative cross-sectional multi-group design

* These data were computed by averaging across all three groups of participants (with schizophrenia, suffering from anxiety and/or depression, and with personality disorders, which were pooled together by the authors.

**TABLE 3:** Overall effect sizes (random-effects model) of all meta-analyses.

Outcome	number of studies *k*	Effect size	Lower limit of the confidence interval	Upper limit of the confidence interval	Significance *p*-value	Variance	Standard error	Total sample size	Sample size of group with schizophrenia	Sample size of control group
Creativity	11	−0.79	−1.09	−0.49	0.000	0.02	0.15	727	285	442
Elaboration	5	−0.47	−0.87	−0.08	0.019	0.04	0.20	291	135	156
Flexibility	7	−0.50	−0.76	−0.24	0.000	0.02	0.13	522	237	285
Fluency	8	−0.83	−1.24	−0.42	0.000	0.04	0.21	519	249	270
Originality	5	−0.61	−0.84	−0.38	0.000	0.01	0.12	322	143	179

A positive effect size indicates that the group with schizophrenia scored higher than the control group, while a negative *d* value indicates that the group with schizophrenia scored lower than the control group.

**TABLE 4:** Heterogeneity analysis presented for each outcome.

Outcome	Cochran’s Q	Degree of freedom	Significance *p*-value	*I* ^2^	*T* ^2^	*T*
Creativity	29.39	10	0.001	65.97	0.16	0.40
Elaboration	8.78	4	0.067	54.45	0.10	0.32
Flexibility	11.65	6	0.070	48.48	0.06	0.24
Fluency	31.76	7	0.000	77.69	0.26	0.51
Originality	3.56	4	0.469	0.00	0.00	0.00

*I*^2^ = 0–24 as negligible heterogeneity; *I*^2^ = 25–49 as low; *I*^2^ = 50–74 as moderate; and *I*^2^ = 75–100 as high heterogeneity.

Analyses of publication bias (e.g., Egger’s test, trim-and-fill procedure) and meta-regression by year of publication were conducted only for the main meta-analysis on overall creativity. These tests were not repeated for the subcomponents (elaboration, flexibility, fluency, and originality) due to the limited number of studies in each sub-analysis and the fact that all included studies—except for one in the flexibility subset by Kucwaj et al. (2023)—were already part of the overall creativity dataset. This approach avoids redundancy and reflects current methodological recommendations against conducting bias analyses on small or overlapping subsets.

Except for the main overall creativity analysis, all other subscore meta-analyses (elaboration, flexibility, fluency, and originality) included fewer than 10 studies (*k* < 10) and should therefore be interpreted with caution. In such small samples, estimates of effect size, heterogeneity, and publication bias may be less stable and statistically underpowered.

### 3.1 Creativity in individuals with schizophrenia compared to healthy controls

**FIGURE 2:**
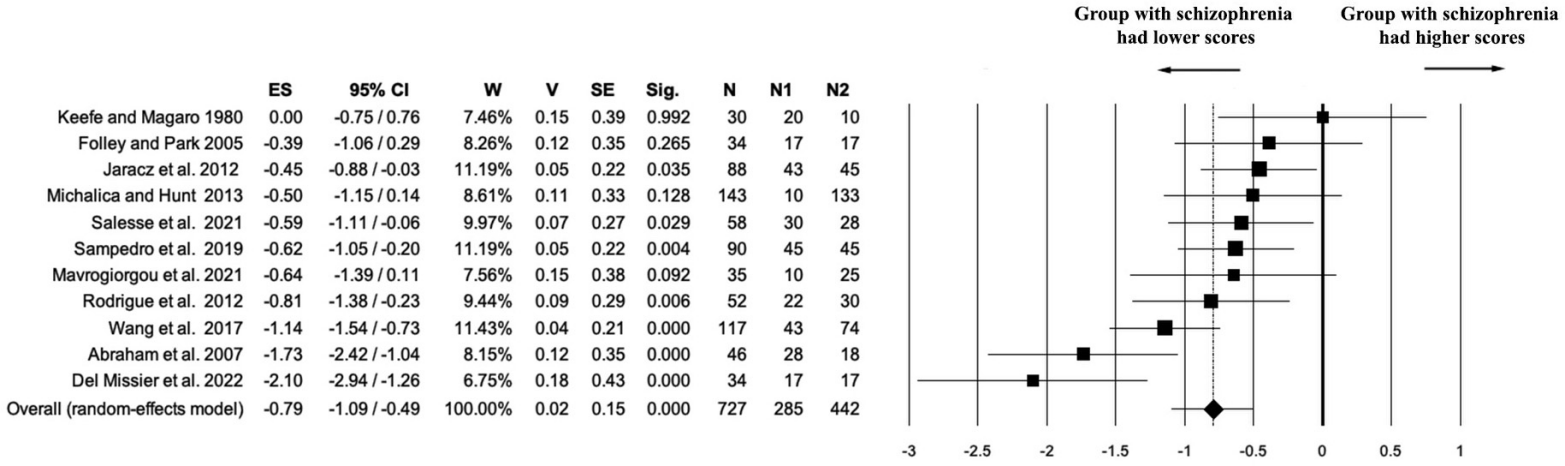
Forest plot of the “creativity” outcome. The meta-analysis yielded a statistically significant negative effect size, indicating lower creativity in the schizophrenia group compared to healthy controls (ES = –0.79).

A total of 11 studies (*k* = 11) directly addressed the question of whether individuals with schizophrenia differ in creativity from healthy controls. The combined sample included 727 participants (*n*1 = 285 patients, *n*2 = 442 controls), with no missing data (n_na = 0). The meta-analysis revealed a statistically significant negative effect size, indicating lower creativity in the schizophrenia group compared to healthy controls (ES = –0.79, 95% CI [–1.09, –0.49], *p* < 0.001). The precision of the effect was supported by low variance (*V* = 0.02) and standard error (SE = 0.15) (see Figure 2).

Heterogeneity was substantial [Q(10) = 29.39, *p* = 0.001; *I*^2^ = 65.97%; *T*^2^ = 0.16; *T* = 0.40], which can be reasonably attributed to the complexity of the creativity construct and the wide heterogeneity in measurement tools used across studies. Moreover, in many cases, we aggregated multiple creativity-related outcomes within individual studies by computing a weighted composite score. While variability in effect size magnitude was observed, it is worth noting that all studies reported effects in the same direction, indicating consistently lower creativity scores in individuals with schizophrenia.

Leave-one-out sensitivity analysis further confirmed the robustness of the findings. Excluding each study in turn resulted in effect sizes ranging from–0.70 to –0.85, all of which remained statistically significant (*p* < 0.001). This indicates that no single study disproportionately influenced the overall result (see Supplementary Table 6 and Supplementary Figure 1). When ordered by year of publication, the cumulative meta-analysis showed that the negative effect size became statistically significant starting from 2005 and remained stable across later studies. Although some recent studies, such as Mavrogiorgou et al. (2021), did not reach individual significance, their effect sizes were consistent in direction and contributed to the overall pattern. This supports the robustness and temporal consistency of the observed association (see Supplementary Table 7).

**FIGURE 3:**
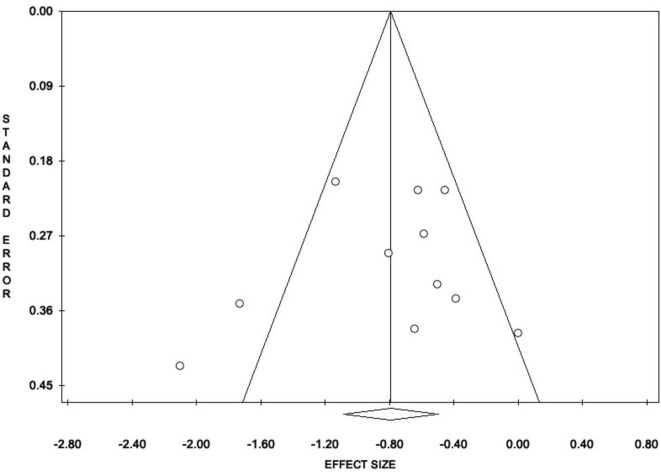
Trim and Fill procedure. Results indicated substantially no risk of bias.

Tests for publication bias revealed no significant concerns. Egger’s regression intercept was non-significant (intercept = –1.01, *t* = –0.46, *p* = 0.654). The Trim and Fill procedure imputed only one potential missing study and yielded a slightly more conservative estimate (Observed ES = –0.79, Estimated ES = –0.86), with both remaining statistically significant (*p* < 0.001) (see Figure 3). The Fail-safe N was 217, far exceeding the tolerance threshold (5k + 10 = 65), indicating that the observed effect is unlikely to be explained by unpublished null results. Finally, a weighted meta-regression using publication year as a predictor (random-effects model) showed no significant trend over time (*k* = 11, *Y* = –39.00 + 0.02x, *p* = 0.508; see Supplementary Figure 2).

### 3.2 Elaboration in individuals with schizophrenia compared to healthy controls

**FIGURE 4:**
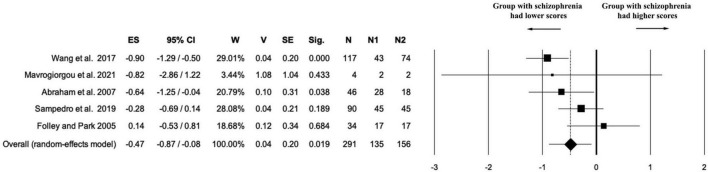
Forest plot of the “elaboration” outcome. The meta-analysis yielded a statistically significant negative effect size, indicating a lower level of elaboration in the schizophrenia group compared to healthy controls (ES = - 0.47).

A total of five studies (*k* = 5) directly addressed the question of whether individuals with schizophrenia differ in elaboration from healthy controls. The combined sample included 291 participants (*n*1 = 135 patients, *n*2 = 156 controls), with no missing data (*n*_na = 0). The random-effects model yielded a small-to-moderate negative effect size in favor of the control group (ES = –0.47, 95% CI [–0.87, –0.08], *p* = 0.019). Variance and precision were acceptable (*V* = 0.04; SE = 0.20), indicating that the estimate is reasonably stable despite the limited number of studies. Overall, these findings suggest that patients with schizophrenia display significantly lower elaboration scores than healthy controls, although the magnitude of the deficit is smaller than that observed for the global creativity index (see Figure 4).

The analysis of heterogeneity revealed moderate between-study variability [Q(4) = 8.78, *p* = 0.06; *I*^2^ = 54.45%; *T*^2^ = 0.10; *T* = 0.32]. Although Cochran’s Q test did not reach conventional statistical significance, this is likely due to the limited number of studies included. The I^2^ value suggests that methodological or measurement differences across studies may have contributed to the observed dispersion.

Sensitivity analysis confirmed the stability of the negative effect on elaboration scores. Excluding individual studies did not change the direction of the effect, and in three out of five iterations the results remained statistically significant. This supports the overall robustness of the finding, although two studies (Abraham et al., 2007; Wang et al., 2017) marginally reduced statistical significance (see Supplementary Table 8 and Supplementary Figure 3).

A cumulative meta-analysis was conducted on the five studies reporting elaboration scores, ordered by year of publication. The results indicated that the negative effect size emerged early (with statistical significance from the third study onward) and remained relatively stable across subsequent studies. Although the limited number of included studies (*k* = 5) reduces the interpretative power of this analysis, the consistency in effect direction supports the robustness of the overall finding (see Supplementary Figure 4).

### 3.3 Flexibility in individuals with schizophrenia compared to healthy controls

**FIGURE 5:**
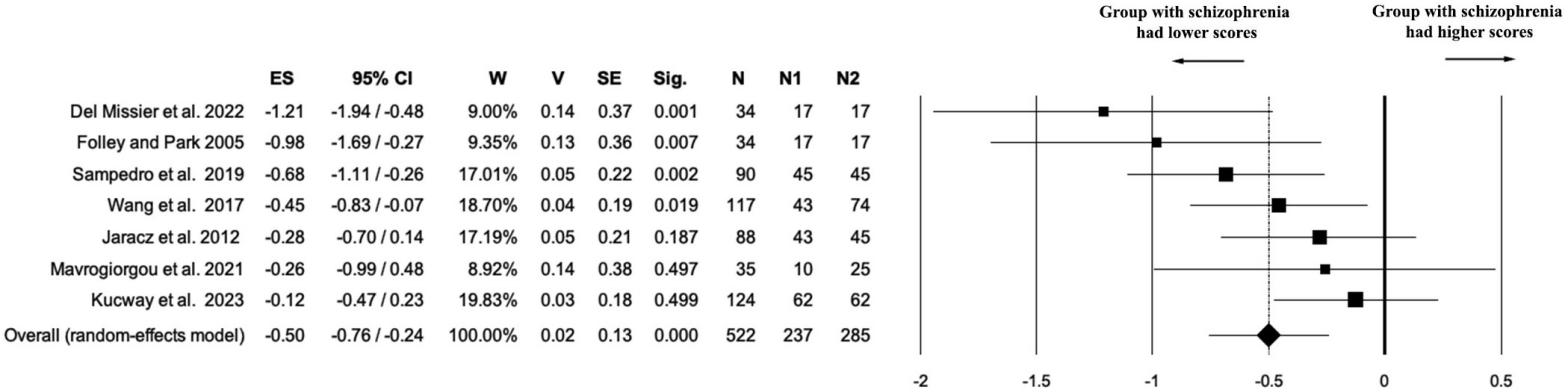
Forest plot of the “flexibility” outcome. The random-effects meta-analysis revealed a statistically significant negative effect size in favor of the control group (ES = –0.50).

Seven studies (*k* = 7) contributed data on the Flexibility component of creativity, including a total of 522 participants (*n*_1_ = 237 individuals with schizophrenia; *n*_1_ = 285 healthy controls; *n*_na_ = 0). The random-effects meta-analysis revealed a statistically significant negative effect size in favor of the control group (ES = –0.50, 95% CI [–0.76, –0.24], *p* < 0.001), indicating that individuals with schizophrenia performed worse on flexibility tasks compared to healthy participants. The effect estimate showed acceptable precision, with a variance of 0.02 and a standard error of 0.13 (see Figure 5).

Heterogeneity was moderate [Q(6) = 11.65, *p* = 0.07; *I*^2^ = 48.48%; *T*^2^ = 0.06; *T* = 0.24], suggesting some variability in effect sizes across studies, possibly due to methodological differences or measurement variability. While the direction of the effect was largely consistent, one study showed a small effect favoring the schizophrenia group, slightly contributing to the overall dispersion.

Sensitivity analysis confirmed the robustness of the effect observed for flexibility. Removing one study at a time (leave-one-out approach) yielded consistently negative and statistically significant effect sizes (range: ES = –0.41 to –0.58, all *p* ≤ 0.002), indicating that no single study disproportionately influenced the overall result (see Supplementary Table 9 and Supplementary Figure 5).

### 3.4 Fluency in individuals with schizophrenia compared to healthy controls

**FIGURE 6:**
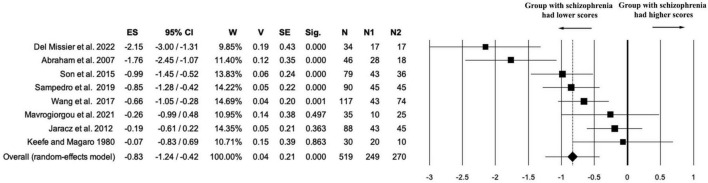
Forest plot for “fluency” outcome. The random-effects meta-analysis revealed a statistically significant negative effect size in favor of the control group (ES = –0.83).

Eight studies (*k* = 8) contributed data on the Fluency component of creativity, for a total of 519 participants (*n*_1_ = 249 individuals with schizophrenia; *n*_2_ = 270 healthy controls; *n*_na_ = 0). The random-effects meta-analysis revealed a statistically significant negative effect size in favor of the control group (ES = –0.83, 95% CI [–1.24, –0.42], *p* < .001), indicating that individuals with schizophrenia scored lower on fluency measures compared to healthy participants. The estimate was associated with moderate precision (*V* = 0.04; SE = 0.21), supporting the reliability of the observed effect (see Figure 6).

Heterogeneity was substantial [Q(7) = 31.76, *p* < 0.001; *I*^2^ = 77.96%; *T*^2^ = 0.26; *T* = 0.51], suggesting considerable variability in effect sizes across studies. This dispersion may be attributed to methodological differences in how fluency was operationalized or assessed, as well as variability in the cognitive profiles of the included samples. While the magnitude of the effect varied, the directionality of the effect was largely consistent across studies.

Sensitivity analysis confirmed the robustness of the fluency effect. Across all iterations, the direction and statistical significance of the effect remained unchanged (ES range: –0.69 to –0.94; all *p* ≤ 0.001), indicating that no individual study disproportionately influenced the overall result. The variation in effect size was moderate, which helps explain the substantial heterogeneity observed in the main analysis (see Supplementary Table 10 and Supplementary Figure 6.

### 3.5 Originality in individuals with schizophrenia compared to healthy controls

**FIGURE 7:**
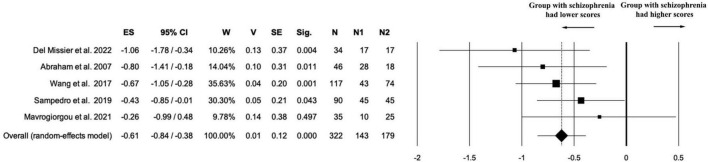
Forest plot of “originality” outcome. The random-effects meta-analysis revealed a statistically significant negative effect size in favor of the control group (ES = –0.61).

Five studies (*k* = 5) contributed data on the originality component of creativity, including a total of 322 participants (*n*_1_ = 143 individuals with schizophrenia; *n*_2_ = 179 healthy controls; *n*_na_ = 0). The random-effects meta-analysis revealed a statistically significant negative effect size in favor of the control group (ES = –0.61; 95% CI [–0.84, –0.38]; *p* < 0.001), indicating that individuals with schizophrenia scored lower on measures of originality compared to healthy participants. The estimate was associated with high precision, as indicated by low variance (*V* = 0.01) and standard error (SE = 0.12) (see Figure 7).

Heterogeneity was negligible [Q(4) = 3.56; *p* = 0.469; *I*^2^ = 0.00%; *T*^2^ = 0.00; *T* = 0.00], suggesting a high degree of consistency across studies in both the magnitude and direction of the effect.

Given the small number of studies (*k* = 5) and the lack of heterogeneity (*I*^2^ = 0.00%), no sensitivity analysis was conducted for the originality subscore.

### 3.6 Moderator analyses

Moderator analyses were conducted for each of the five creative outcomes—creativity, elaboration, flexibility, fluency, and originality—using meta-regression models. Overall, the results revealed limited evidence of moderating effects. Among the variables tested, only the mean age of the experimental group emerged as a statistically significant moderator. Specifically, older age was associated with lower scores in creativity (β = –0.05; *p* = 0.04), flexibility (β = –0.04; *p* = 0.035), and fluency (β = –0.07; *p* = 0.026), suggesting a potential decline in these dimensions of creativity with increasing age in patients.

In contrast, the publication year, the mean age difference between patients and controls, the proportion of males in the experimental group, as well as the mean years of education and the educational gap between groups did not significantly moderate the results across any outcome (all *p* > 0.13). These findings suggest that publication-related and demographic variables, except for patient age, played a minimal role in accounting for variability in effect sizes (see Supplementary Table 11).

Although nearly all studies reported that participants with schizophrenia were taking antipsychotic medication, only a few specified the medication class, and even fewer provided quantitative dosage information (e.g., CPZE or haloperidol equivalents). Due to the limited and heterogeneous nature of the available data, no moderator analysis based on medication type or dosage could be conducted.

## 4 Discussion

### 4.1 Interpreting the data: deficit in normative creativity

This meta-analysis offers an in-depth synthesis of the available evidence on creativity in individuals with schizophrenia compared to healthy controls. In all five domains examined—overall creativity, elaboration, flexibility, fluency, and originality—the results consistently indicate a disadvantage for patients with schizophrenia. The main analysis on overall creativity showed a robust and statistically significant effect, indicating that such patients tend to perform worse in creative tasks compared to healthy participants. This trend is also reflected in the specific subdomains, where significant deficits emerge, albeit with varying effect sizes.

Particularly relevant is the consistency in the direction of effects: nearly all studies report lower scores for patients compared to controls. The convergence of results across different tools and experimental designs strengthens the conclusion that creativity—understood here as the ability to produce original and effective solutions to specific problems—is generally impaired in schizophrenia. These results are consistent with what was reported by Acar et al. (2018), suggesting that the narrative outlined by quantitative research on creativity in schizophrenia has remained substantially stable over the past 8 years.

Although these results confirm previous conclusions, the current data suggest that creative deficits are both broad and stable, affecting various components of creative cognition. The consistent directionality and overall coherence of the data, despite variations in effect size across studies and domains, provide a reliable picture with significant implications for both theory and clinical practice.

### 4.2 The phenomenological lens: loss of common sense and the “existential diet” of treatment

The consistency of these deficits, however, does not exclude the well-documented presence of exceptional creative manifestations in some patients with schizophrenia. These cases should not be considered mere statistical outliers, as Dietrich (2014) warns when criticizing the neglect of base-rate in creativity research. On the contrary, they could reflect complex individual trajectories that are still poorly investigated. Indeed, Sass’s perspective on the limitations of utilitarian tests in assessing creativity in patients with psychopathological disorders deserves greater attention. Morgenthaler (1992), for example, interprets the art of Adolf Wölfli not as a simple side effect of psychosis, but rather as a creative process that arises precisely from the psychotic experience: an attempt at symbolic recomposition of the Self through imagination. Through this reinterpretation, the psychiatrist highlights the distinctive expressive power of his patient’s art.

If psychopathology offers an original perspective on the world—and thus a potentially creative one—why do the data systematically confirm lower performance in standardized tests? Moderator analyses indicated that most demographic and methodological variables do not account for the observed differences. Only one variable proved to be significant: the age of the patients. Older individuals with schizophrenia showed greater deficits, particularly in the domains of flexibility and fluency. Neither the age of healthy participants nor the age gap between experimental groups was associated with creative performance, suggesting that the observed cognitive decline may be specific to patients with schizophrenia. This finding is consistent both with studies indicating the absence of a negative relationship between age and creativity in the healthy population (Binnewies et al., 2008; Acar et al., 2020) and with those showing a general cognitive decline in patients with schizophrenia (McCutcheon et al., 2023).

A possible explanation lies in the duration of cognitive deterioration: older patients may have experienced such a decline for a longer period. A recent meta-analysis identified persistent cognitive dysfunction as a transdiagnostic feature of psychiatric disorders (Abramovitch et al., 2021). It is also likely that older patients have been taking medications for a longer time: with the exception of Michalica and Hunt (2013), all the studies in our sample reported that patients were at least being treated with antipsychotics, and in one case (Del Missier et al., 2022), other medications were also reported.

Recent evidence suggests that prolonged use of antipsychotics—both typical and atypical—may accelerate cognitive decline. An ecological study by Husa et al. (2017) found that greater cumulative exposure to antipsychotics was associated with poorer cognitive outcomes in middle age. Moreover, recent triple-blind randomized placebo-controlled trials (Allott et al., 2023, 2024) have suggested that second-generation antipsychotics, which were prevalent in our sample, may impair crucial cognitive areas such as verbal memory and learning. A review of qualitative studies on subjective perception of antipsychotic’s effects shows that, alongside the dampening and clinically desired effects of antipsychotics (reduction in the intensity of voices, decreased intrusiveness of delusional thoughts, greater calmness and stability, and improved sleep), frequently patients experience a form of cognitive slowing and emotional blunting, which frequently compromise creativity, imagination, and the ability to concentrate (Thompson et al., 2020). Indeed, a double-blind, placebo-controlled, crossover study in 50 healthy volunteers showed that amisulpride and aripiprazole (widely used antipsychotics for the treatment of schizophrenia and other psychotic disorders, effective in reducing delusions, hallucinations, and affective instability) do not produce global sedation or changes in vigilance, but are associated with a statistically significant slowing of working-memory response times compared with placebo (Osugo et al., 2025). However, the issue remains unresolved, as a substantial body of the literature shows that the cognitive deficits typically associated with schizophrenia are already present at the first episode, before patients have begun antipsychotic treatment (Lee et al., 2024).

Beyond the pharmacological effects, which are still being elucidated, the results of the present meta-analysis can also be read through a phenomenological lens. Sass and Parnas (2003) describe schizophrenia as a disruption of ipseity, marked by hyper-reflexivity, loss of self-affection, and disconnection from the lived world. From this perspective, psychiatric treatment inevitably aims to correct this deviation from shared reality, but in doing so, it may also diminish or suppress the patient’s unique subjective experience, from which creativity may originate.

Thus, the observed deficits in creativity should not be interpreted as a primary symptom of the disorder itself, but rather a potential side effect of the psychiatric treatment’s necessity to contain the patient’s subjective experience, insofar as it is not socially shared or intelligible. The imposition of this “existential diet”, if prolonged over time, may impair access to one’s own Self and thus the ability to produce original and effective responses in tests.

It is important to underline that this line of reasoning should not be misunderstood as a criticism of psychiatric treatment per se. Antipsychotics remain a cornerstone in the treatment of schizophrenia (Correll et al., 2022), offering relief from severe symptoms and improving quality of life and daily functioning (Sampogna et al., 2023). What we are trying to underline here is the importance of fully understanding, in all possible nuances, the effects that treatments have on the cognitive processes of human beings in general. In line with this perspective, Fasciolo Maschião and Messas (2025) propose a new research program called *phenomenological psychopharmacology*, aimed at investigating how an exogenous substance may bring about changes in lived experience, not merely through the reporting of symptoms, but rather through an analysis of modifications in the pre-reflective structure of subjectivity, thus addressing the qualitative transformation of the structures of experience following the intake of exogenous substances.

### 4.3 Cognitive performance in delusion-resonant tasks

From a philosophical point of view, if schizophrenia involves—as Sass (2003) suggests—a reconfiguration of the structures of subjectivity through hyper-reflexivity and detachment from the lived world, then any clinical intervention aimed at stabilizing the Self will inevitably entail its modulation. Yet, it is precisely in the dynamic tension between affective interiority and symbolic externalization that creativity is born. While stabilizing or normalizing the Self may be necessary, it can inadvertently lead to a sort of “existential diet” that limits its expressive and regenerative capacities.

The language of the schizophrenic patient, for example, often appears incomprehensible and is classified as a formal thought disorder. However, qualitative studies have repeatedly highlighted how such an anomaly may conceal a sophisticated internal logic and a creative use of language (Pennisi, 1998; Cardella, 2017; Cardella and Falzone, 2021; Falzone et al., 2023; Pennisi, 2022). This would not so much be a dysfunction, but rather a different way of meaning-making. What is lacking, in these cases, is the sharing of a common experiential world, a shared sense of the world (Blankenburg, 1971; Binswanger, 1956; Minkowski, 1927), whose loss may give rise to forms of compensatory hyper-rationalism.

This interpretation is distant from the one offered by normative quantitative tests, which do not take the experiential context into account. Recent studies have challenged this view: Gangemi et al. (2020), for example, showed that patients with schizophrenia can outperform controls in syllogistic tasks when the content of the task resonates with their delusional experience. The same applies to pragmatic skills, often considered deficient (Li et al., 2017; Bambini et al., 2016), but which qualitative studies have reinterpreted differently (Pennisi and Bucca, 2025; Bucca, 2022).

Schizophrenic creativity, too, in its most evocative, symbolic, and self-expressive aspects, has often been described by the qualitative literature as an attempt at recomposing the Self through imagination (Pennisi, 1998; Bucca, 2013, 2015, 2019, 2024). It is therefore not surprising that the historical figures of the “mad geniuses” have fascinated the collective imagination so much. But this type of creativity, rooted in lived subjectivity, escapes the standardized metrics of tests. In our view, studies on creativity in schizophrenia that do not take into account the subjectivity described by the phenomenological and philosophical literature risk not being fully ecological. If the patient has lost common sense, it is unlikely that they will find motivation in performing a generic task imposed from the outside. In light of these considerations, we believe that when the Self is contained in its expressive intensity, in its affectivity, or in its imaginative freedom, creativity may become an unintended collateral victim. The challenge is not to forgo treatment, but to accompany it with a careful attention to what might be lost: the symbolic depth and uniqueness of the subject’s lived experience.

Finally, it is important to remember that numerous forms of creative expression have shown tangible benefits for psychological wellbeing (Acar et al., 2020; Ciufalo et al., 2024). From this perspective, an interdisciplinary approach—including clinical, cognitive, and philosophical aspects—could offer valuable tools not only for better symptom management, but also to promote a richer, more authentic, and humanly sustainable quality of life.

### 4.4 Limitations and future research directions

Since we adopted a highly conservative approach to study inclusion, our final sample was relatively small. In particular, the meta-analyses on flexibility, fluency, elaboration, and originality included fewer than 10 studies (*k* < 10) therefore, the results in these domains should be interpreted with caution. Further research in this area is needed to strengthen the evidence base.

Given that the moderator analyses are only correlational, future studies would benefit from directly investigating the relationship between age and creativity in schizophrenia using tests similar to those analyzed in this study (e.g., TTCT and related tools). Where possible, such studies should also control for the relationship between prolonged medication use and lower performance on standardized creativity assessments.

The study of creativity in schizophrenia must be approached from an interdisciplinary perspective that integrates phenomenological and qualitative approaches (such as analytical studies conducted on the preserved works of hospitalized patients not treated with antipsychotics; see Cardella, 2011; Pennisi, 2011; Pennisi, 1998) alongside quantitative methods. In fact, another limitation of this study is the lack of data on the antipsychotic medication used (including dose, duration, agent, and anticholinergic burden).

The ultimate goal should be to develop more ecologically valid assessment tools—methods capable of capturing the existential reality experienced by patients, which, unfortunately, is often inevitably restricted by psychiatric, psychological, and pharmacological treatments aiming to return them to a shared sense of reality.

Finally, another promising line of research is the one on creativity in relatives of people with schizophrenia; i.e. recently has been showed that creativity and schizophrenia share a matrix of vulnerability that primarily involves disturbance of the minimal self, loss of natural self-evidence, hyper-reflexivity, and alteration of imagination and sense of reality (Parnas et al., 2019a)

## 5 Conclusion: toward a more integrative perspective

In this meta-analysis, we addressed the longstanding paradox between a substantial body of qualitative literature—which demonstrates the creative potential expressed through both artistic and other forms of production by individuals with schizophrenia—and the contrasting quantitative findings that consistently report poor performance on standardized creativity tests. Drawing inspiration from a previous meta-analysis (Pennisi et al., 2021) that revealed a distinctive but non-deficient creativity profile in individuals with autism (based on the subcomponents of creativity: fluency, flexibility, elaboration, and originality), we applied a similar analytic framework to the literature on schizophrenia and creativity. Unlike in autism, however, our results did not reveal a divergent but intact creative profile. On the contrary, individuals with schizophrenia performed significantly worse than healthy controls not only in overall creativity scores but also across all four subcomponents. These findings reinforce the view of a consistent creativity impairment in schizophrenia as measured by conventional testing tools.

Yet a notable insight emerged from our moderator analysis. Among all variables examined, only the age of the patients significantly moderated creative performance. Neither the age of control participants nor the age difference between groups showed any effect. To interpret this result, we adopted a phenomenological perspective.

Phenomenological accounts describe schizophrenia as a fundamental disruption in the structure of subjectivity—a detachment from the shared, intersubjective world and a retreat into a self-generated reality. Psychiatric treatment, whether pharmacological or psychological, is thus oriented toward restoring a connection with common reality. However, in doing so, treatment may also constrain the patient’s idiosyncratic inner world, including the creative expressions it enables.

Indeed, clinical and anecdotal observations often report striking creative output in individuals with schizophrenia when the content of their creations aligns with their delusional beliefs. This suggests that standardized assessments may systematically underestimate the creative potential of these individuals precisely because such instruments are not attuned to the thematic and affective significance of their inner world.

We therefore propose that the apparent discrepancy between qualitative and quantitative findings may not reflect a contradiction in data, but rather a consequence of how creativity is measured. It may be interesting, in this case, to recall the case of the 38 year-old painter reported by Minkowski (1933). Before the emergence of the pathology, the man was a prolific painter who worked with zeal. When, however, the disorder became clinically manifest, the painter reported that every movement of his arm during painting was accompanied by the voices, and this prevented him from completing the paintings. He was not able to see the faces of the entities that produced the voices, but he made an effort to imagine them. His figurative production, therefore, had not become extinguished, but had shifted from the plane of the artistic work to that of hallucinatory figuration. Minkowski also reports that, during the years of the pathology, the painter nevertheless managed to bring one painting to completion, but this painting now turned out to be completely different from the previous ones, no longer belonging to the same continuity of sense as the others. In the following years, the patient acquired the habit of obsessively transcribing in notebooks the dialogues with the voices he heard, in search of a formal structuring for that perception. The point, therefore, is not that the painter had lost his creative capacities, but that the entire field of sense of his experience had reorganized itself around the pathology. His creative attitude, now having to organize a different content, had shifted toward different forms (namely, those of the transcription of the voices). But if the whole production of sense shifts to the contents of the pathology, there is no longer any space for the social demands of standardized tests, such as the TTCT, which was developed, for example, to select innovative minds functional to a productive and strategic system for society (Torrance, 1966). Standardized tests, by design, often exclude content that deviates from normatively accepted frameworks. As a result, the imaginative productivity of patients—especially when shaped by personal or psychotic experiences—may be filtered out or pathologized.

We hypothesize that the diminished creativity observed in quantitative studies may represent a side effect of prolonged treatment: a “diet” imposed on the patient’s imaginative life in favor of restoring alignment with the shared world. This hypothesis is partially supported by retrospective qualitative studies conducted on hospitalized patients who were not treated with antipsychotics, or with pharmacological treatments comparable to those taken by participants of the studies included in this meta-analysis. These studies clearly show that patients still exhibit a marked capacity for imaginative creation and for producing, within the context of their delusions, complex narratives, paintings, or sculptures (Cardella, 2011; Pennisi, 2011; Pennisi, 1998; Bucca, 2015). If creativity were assessed using tools that are sensitive to the subject’s own thematic and expressive interests—even those reflecting delusional content—we hypothesize that individuals with schizophrenia would display creativity profiles comparable to, or even exceeding, those of healthy controls.

In light of this study, we propose two main directions for future research. On the one hand, it would be valuable to investigate, within longitudinal designs, differences in creative performance in drug-naïve patients before and after the initiation of treatment. On the other hand, research should aim to develop and adopt instruments that assess not so much creative performances, as the lived experience of creativity itself. A promising attempt in this direction is the Examination of Anomalous Fantasy and Imagination (Rasmussen et al., 2018), a semi-structured interview designed to explore whether patients experience changes in the way they themselves experience and relate to their imagination. A markedly different, yet occasionally adopted, approach in scientific literature on creativity consists in statistically examining the prevalence of individual with psychiatric diagnosis or relatives of individuals with diagnosis, with professions typically regarded as highly creative (musicians, mathematicians, painters, academics, etc.) (i.e., Kyaga et al., 2011, 2013; Parnas et al., 2019a,b). Despite important and methodological biases (e.g. for the profession *academics* selective access to academia and the indirect nature of academic status as a proxy of creativity), large-scale population studies provide highly informative indications, even if not definitive, and should therefore be considered as complementary to other methodological and theoretical approaches.

## Data Availability

The original contributions presented in this study are included in this article/Supplementary material, further inquiries can be directed to the corresponding authors.
