# Correction: A meta-analysis and systematic review of creativity in schizophrenia: toward an ecological understanding integrating clinical and philosophical perspectives

**DOI:** 10.3389/fpsyg.2026.1835392

**Published:** 2026-04-07

**Authors:** Paola Pennisi, Federica Longo, Sara Alfia Nicotra, Mohammad Ali Salehinejad, Carmelo Mario Vicario, Alessandra Maria Falzone

**Affiliations:** 1 Department of Adult and Childhood Human Pathology “Gaetano Barresi”, Università degli Studi di Messina, Messina, Italy; 2 Department of Cognitive Sciences, Psychology, Education, and Cultural Studies, Università degli Studi di Messina, Messina, Italy; 3 Department of Child and Adolescent Psychiatry, Psychosomatics and Psychotherapy, Faculty of Medical, RWTH Aachen University, Aachen, Germany; 4 Cognitive Neuroscience Laboratory, Department of Cognitive Sciences, Psychology, Education, and Cultural Studies, Università degli Studi di Messina, Messina, Italy

**Keywords:** creativity, ecological assessment, mad-genius hypothesis, phenomenology, schizophrenia, originality, psychopathology

An **Acknowledgments** statement was erroneously missing from the published article. The full statement is below:

## Acknowledgments

This work is a product of the research carried out within the PRIN 2022 SACRe-D project, “*Schizophrenia, Autism and the Myth of Creativity. An Interdisciplinary Perspective on Psychopathological Expression and its Digitalization*,” identification code PRIN_2022PFSJNW_002, CUP J53D23008240006.

The original version of this article has been updated.

