# Supplementary material for: A meta-analysis and systematic review of creativity in schizophrenia: toward an ecological understanding integrating clinical and philosophical perspectives
Source: Front Psychol. 2026 Mar 4;17:1658295. doi: 10.3389/fpsyg.2026.1658295 (PMC12997125; doi:10.3389/fpsyg.2026.1658295)
Supplement: Supplementary file 1 [file Data_Sheet_1.pdf]

Supplementary Materials

§S1. Notes on coding of data

Kucwaj et al. (2023) reported data in education in a different format from the one used by us. We converted their values into "years of education". It will follow the procedure for conversion we used:

Methodology for Conversion

First Group (Mean = 2.5, SD = 1.21, Range = 1–5)

1. Mean Years of Study: The reported mean value of 2.5 falls between vocational school (2 = 7 years) and high school (3 = 9 years).
2. Standard Deviation (SD): The reported SD of 1.21 was scaled to reflect the differences in years between education levels. The average difference between adjacent levels (e.g., 7–4, 9–7, etc.) was approximately 4.33 years.
3. Range in Years: The reported range of 1–5 was converted as follows:
   - Minimum (1): Primary school = 4 years.
   - Maximum (5): Higher education completed = 14 years.
   - Range: 4–14 years.

Second Group (Mean = 4.1, SD = 1.12, Range = 1–5)

1. Mean Years of Study: The reported mean value of 4.1 is close to the level 4 (student = 12 years).
2. Standard Deviation (SD): The SD of 1.12 was similarly scaled.
3. Range in Years: The range of 1–5 was converted as:
   - Minimum (1): Primary school = 4 years.
   - Maximum (5): Higher education completed = 14 years.
   - Range: 4–14 years.

Mavrogiorgou et al. (2021) reported data in education in a different format from the one used by us. We converted their values into "years of education". It will follow the procedure for conversion we used:

The data represent the years of education for a group of 25 patients distributed across three educational grades. The years of education were calculated as follows:

- Lower grade = 4 years.
- Middle grade = 8 years.
- Upper grade = 12 years.

The study did not report the single group mean age, SD, and range. To estimate the mean age, standard deviation (SD), and range for the two subgroups (schizophrenia and affective disorders), the following steps were undertaken based on the data available:

1. **Mean Age**:

   The mean age for each subgroup was estimated by summing the average age of onset and the average illness duration. For the schizophrenia group, the average age of onset was combined with the mean illness duration, providing an estimate of the current average age for that group. The same method was applied to the affective disorder group.

2. **Standard Deviation (SD)**:

   The SD for each subgroup was estimated by combining the variability in the age of onset and the illness duration. This was achieved by considering both components as independent contributors to the total variability in age. The resulting SD represents the combined influence of these two sources of variability for each subgroup.

3. **Age Range**:

   The range for each subgroup was approximated by taking two standard deviations (approximately 95% of the data under a normal distribution) above and below the mean age. However, these estimates were constrained by the overall range of the entire sample (18–75 years) to ensure consistency with the data provided.

By applying these steps, the mean age, SD, and estimated range for each subgroup were derived, as detailed in the results section. These estimates provide an approximation of the demographic characteristics of the subgroups within the sample, given the available data.

Kyaga et al. (2011). Although the exact number of matched controls and relatives is not clearly specified in the study, it is stated that each individual with a psychiatric diagnosis, as well as each of their relatives, was matched with ten controls. Based on this design, approximately 3 million controls can be estimated for the 301,457 individuals diagnosed with schizophrenia, bipolar disorder, or unipolar depression. Assuming an average of four first- to third-degree relatives per case, we estimate that over 15 million matched controls were included in total.

Hozo, S. P., Djulbegovic, B., and Hozo, I. (2005). Estimating the mean and variance from the median, range, and the size of a sample. *BMC medical research methodology*, *5*, 1-10.

Salesse et al. (2021). In this study, participants' educational attainment was reported using a categorical scale ranging from 1 to 6. As the unit of measurement was not explicitly defined, we inferred that the authors likely adopted a standard 6-point educational coding system commonly used in clinical research. This system is typically mapped to approximate years of education as follows:

Score, Educational Level, Estimated Years

Score 1, No formal education, Estimated Years 0–4

Score 2, Primary education, Estimated Years 5

Score 3, Lower secondary education (middle school), Estimated Years 8

Score 4, Upper secondary education (high school diploma), Estimated Years 13

Score 5, Bachelor's degree (or equivalent), Estimated Years 16

Score 6, Master's/Doctoral degree, Estimated Years 18–21

§S2. Notes on outcomes

Following the methodological guidance by Borenstein et al. (2021), when a primary study reported two distinct scores for the same outcome, we computed a combined value by averaging the two means and estimating the pooled standard deviation (normally assuming $r = 0.05$).

Abraham et al. (2007). To compute a single composite score representing the construct of creativity, defined as the ability to find original and effective solutions to a specific problem, we combined five creativity-related scores reported by Abraham et al. (2007). These included two subscores from the *Creative Imagery Test* (Practicality and Originality), two subscores from the *Alternate Uses Test* (Fluency and Uniqueness), and one score from the Constraints of Examples task.

We first calculated composite scores separately for the Creative Imagery and Alternate Uses tests. For each of these pairs of subscores, we computed the pooled mean and standard deviation, assuming an intercorrelation of $r = 0.7$. This estimate was based on their shared modality and on previous literature reporting moderate to strong correlations between Fluency and Uniqueness in the context of divergent thinking (e.g., Silvia et al., 2008).

Once these intermediate composite scores were obtained, we combined them with the Constraints of Examples score to generate a global creativity index. Because the Constraints of Examples task differs in modality (drawing-based rather than verbal), we applied a more conservative correlation estimate of $r = 0.3$ between this test and each of the two verbal composite scores.

To calculate *originality* we used the subtest Uniqueness of the *Alternate uses Test* and the test Originality of the *Creative Imagery*. Given that these measures assess the same construct but through different modalities (written/verbal vs. imagined/drawn output), we assumed a moderate correlation of $r = 0.3$. Following the procedure described by Borenstein et al. (2021), we calculated the pooled mean and standard deviation using this correlation coefficient.

All pooled means and standard deviations were calculated using the method described by Borenstein et al. (2021).

Del Missier et al. (2022). In order to compute a composite score representing performance on the Alternate Uses Test (AUT), we combined four subscores: Fluency, Originality, Peak Originality, and Feasibility. All of these indicators were derived from the same participant responses and share the same modality (verbal ideation). In line with common methodological practice and based on previous studies reporting moderate to high intercorrelations among these dimensions (e.g., Silvia et al., 2008), we assumed a correlation of $r = 0.7$ among the subscores.

To create a composite score for the outcome “originality”, we combined two sub-scores from the Alternate Uses Test: the average originality score and the peak originality score. Given that both indices are derived from the same task and assess closely related aspects of creative output, we assumed a strong correlation of $r = 0.7$ between them.

Folley and Park (2005). To derive a single composite score from the two subcomponents of the Novel Divergent Thinking Test (Singular Uses and Combinatory Uses), we computed the mean and pooled standard deviation of the two subscores. Since no empirical estimate of the correlation between these two measures was reported in the original study, we conservatively assumed a correlation of $r = 0.5$, as recommended by Borenstein et al. (2021) when no better estimate is available.

Keefe and Magaro (1980). Since the aim of our meta-analysis was to examine group-level differences between individuals with schizophrenia and healthy controls, regardless of subtype, we combined the data from the paranoid and non-paranoid schizophrenia subgroups.

To compute a single composite score representing the construct of creativity, we combined two sub-scores obtained from the same divergent thinking task: the number of responses (fluency) and the graded level. Given that both scores were derived from the same test, relied on the same verbal production modality, and targeted closely related aspects of idea generation, we assumed a correlation of $r = 0.7$ between them. This assumption is consistent with prior literature reporting moderate-to-strong associations between measures of fluency and creativity (e.g., Silvia et al., 2008).

Kucway et al. (2023); Mavrogiorgou et al. (2021). For studies that reported non-significant group differences without specifying the exact p-value, we conservatively imputed a p-value of 0.50 for statistical synthesis.

Michalica and Hunt (2013). We excluded the group of professional artists from our analysis, as we considered it more appropriate to compare the creative performance of patients with controls who, like the patients, did not engage in daily creative work as part of their profession. Therefore, we pooled the student group with the age-matched control group (originally matched to the artists). This was a more conservative choice, aiming to avoid potential confounds related to occupational exposure to creative tasks.

Rodrigue et al. (2012). In the study by Rodrigue et al. (2012), although the Abbreviated Torrance Test for Adults (ATTA) was administered, subscale scores for fluency, originality, flexibility, and elaboration were not explicitly reported. The reported results focused on a comparison between patients with schizophrenia and a control group defined as “low S Scale” individuals, i.e., individuals without psychotic traits. However, due to the absence of precise numerical values for each subscale, we adopted a conservative approach and excluded this study from subscale-specific analyses. This decision aligns

with methodological standards recommended in the Cochrane Handbook, which discourage inferring quantitative estimates in the absence of clearly reported outcome data, particularly for meta-analytic purposes.

Sampedro et al. (2019). To compute composite scores for the outcomes originality, flexibility, and fluency, we combined two test scores for each construct reported in the primary studies. In each case, we assumed a correlation of r = 0.5 between the paired measures. This choice reflects the fact that the measures assess the same underlying construct but rely on different sensory or expressive modalities (e.g., verbal vs. visual tasks). Following the methodological recommendations by Borenstein et al. (2021), we computed pooled means and standard deviations using the formulas for combining dependent means when the correlation is estimated.

Son et al. (2015). To compute a single composite score for the construct of fluency, we combined four subtests: two verbal fluency scores (Verbal-C and Verbal-L), one design-based fluency score, and one idea-based fluency score.

First, we averaged the two verbal subtests (Verbal-C and Verbal-L), assuming a correlation of $r = 0.07$ due to the shared modality but different test formats. This low correlation value reflects the moderate relation often found in the literature among verbal fluency components, even when derived from the same overarching test.

Next, we computed the final composite fluency score by averaging the verbal composite, the design fluency score, and the idea fluency score. Given the different modalities (verbal, visual, ideational) but the shared theoretical construct, we assumed a correlation of $r = 0.5$ among these three components. This approach is consistent with previous practices in meta-analytic synthesis when combining multiple indicators of a unified construct with partially overlapping measurement features.

Wang et al. (2017). To compute the overall creativity score, we first created composite scores for each individual task. For the Alternative Uses Task (AUT), we combined the subtest scores assuming an inter-correlation of $r = 0.7$. The same correlation was assumed for the three subtests of the Figure Completion Task, as well as for the two subtests of both the Tangram Construction Task and the Story Generation Task. These four task-level composite scores were then aggregated into a single global creativity score, assuming an inter-task correlation of $r = 0.5$, as they capture creative performance through different modalities.

For the originality and elaboration component specifically, we computed a composite score based on all originality subscores and elaboration subscores across tasks, using an assumed correlation of $r = 0.5$, given that they measure the same construct through different formats.

Finally, the two non-psychiatric participant groups (low schizotypy and high schizotypy) were treated as a single control group by calculating weighted means of the relevant scores.

§S3. Comprehensive results from the systematic review

Table-Suppl1. Data coding for creativity, intended as the ability to find original and effective solutions to a specific problem.

**Study**	**Definition**	**Index for the meta-analysis**
Abraham et al. 2007	Manipulation of figures<br>How functionally and usable the invention is<br>+<br>How unusual and unique the invention is<br>+<br>Imagine and draw a new and different toy with specific features<br>+<br>Verbal written<br>The number of acceptable solutions when participants was asked to generate as many uses as possible for three common objects<br>+<br>The infrequency of given solutions	Score "practicality" at the *Creative imagery test*<br>+<br>Score "originality" at the *Creative imagery test*<br>+<br>*Constraints of examples*<br>+<br>Score "fluency" at the *Alternate uses test*<br>+<br>Score "originality" at the *Alternate uses test*
Del Missier et al. 2022	Verbal written<br>The degree to which the proposed use of the object could represent an actual use<br>+<br>The number of acceptable solutions<br>+<br>Mean of originality ratings<br>+<br>Originality ratings of the most original responses	Score Feasibility at the *Alternate uses test*<br>+<br>Score Fluency at the *Alternate uses test*<br>+<br>Score average originality at the *Alternate uses test*<br>+<br>Score Peak Originality at the *Alternate uses test*
Folley and Park 2005	Verbal<br>Ability to generate uses for real objects (familiars or unfamiliar)	Singular uses + combinatory uses at the *Divergent Thinking Task*
Jaracz et al. 2012	Drawings, verbal and numeric<br>Flexible generation of ideas, imagination and skill for consideration of many possible solutions measured by figural, verbal and numeric inventiveness tasks	Total score at the *Berlin Intelligence Structure Test*
Keefe and Magaro 1980	Verbal<br>Total number of responses<br>+<br>Assessed creativity (creativity scored for appropriateness of response and originality)	Composite score of *Alternate uses test*
Mavrogiorgou et al. 2021	The ability to find original and effective solutions to a specific problem	TTCT

Michalica and Hunt 2013	Self-rated measure of creativity and creative hobbies	Self-rated creativity
Rodrigue et al. 2012	The ability to find original and effective solutions to a specific problem	*Abbreviated Torrance Test* for Adults
Sampedro et al. 2019	The ability to find original and effective solutions to a specific problem	TTCT + Unusual Uses subtest
Wang et al. 2017	The ability to find original and effective solutions to a specific problem	AUT+ Figure completion + Tangram construction + story generation

Table-Suppl2. Data coding for elaboration, intended as the level of detail reached by each answer.

**Study**	**Definition**	**Index for the meta-analysis**
Abraham et al. 2007	Imagine and draw<br>Bilateral symmetry of form, appendages (legs, arms, wings, tail), sense organs (eyes, mouth, nose, ears), atypical appendages, and atypical sense organs<br>+<br>Input visual, output verbal<br>Number of responses that included a use for at least two objects within the stimulus set when was asked to participants to imagine more uses for some set stimuli	Score at *Conceptual expansion*<br>+<br>Combinatory uses of *Divergent thinking task*
Mavrogiorgou et al. 2021	Level of detail reached by each answer	TTCT
Sampedro et al. 2019	Level of detail reached by each answer	TTCT
Wang et al. 2017	Level of detail reached by each answer	Elaboration at figure completion + Tangram construction

Table-Suppl3. Data coding for flexibility, intended as the ability to produce semantically different ideas.

**Study**	**Definition**	**Index for the meta-analysis**
Del Missier et al. 2022	Words were read on a screen and output was verbal<br>Participants were presented with three words and instructed to find another word that could be matched with each of the three-stimulus word (% of correct responses)	RAT Accuracy score
Folley and Park 2005	Verbal written<br>Participants were presented with three words and instructed to find another word	RAT Accuracy score

	that could be matched with each of the three-stimulus word	
Jaracz et al. 2012	Verbal<br>The ability to find alternative uses of a given object	AM score at the *Berlin Intelligence Structure Test*
Kucwaj et al. 2023	Verbal written<br>Participants were presented with three words and instructed to find another word that could be matched with each of the three-stimulus word<br>+<br>Verbal written<br>Insight problems	RAT Accuracy score<br>+<br>Proportion of problems solved correctly
Mavrogiorgou et al. 2021	The ability to produce semantically different ideas	TTCT
Sampedro et al. 2019	The number of different categories used	TTCT + Unusual Uses subtest
Wang et al. 2017	Number of different categories responses fell into	Flexibility at *Alternate uses test*

Table-Suppl4. Data coding for fluency, intended as the quantity of responses produced for each request.

**Study**	**Definition**	**Index for the meta-analysis**
Abraham et al. 2007	Verbal written<br>The number of acceptable solutions when participants was asked to generate as many uses as possible for three common objects	Score "fluency" at the *Alternate uses test*
Del Missier et al. 2022	Verbal written<br>The number of acceptable solutions when participants was asked to generate as many uses as possible for three common objects	Score "fluency" at the *Alternate uses test*
Jaracz et al. 2012	Verbal<br>Generate as many sentences as possible, containing three given nouns<br>+<br>Figural<br>Create as many objects as possible from one figure	MA score at the *Berlin Intelligence Structure Test*<br>+<br>ZF score at the *Berlin Intelligence Structure Test*
Keefe and Magaro 1980	Verbal	N° of responses at the *Alternate uses test*

	The number of solutions proposed from participants was asked to generate as many uses as possible for three common objects	
Mavrogiorgou et al. 2021	Quantity of responses produced for each request	TTCT
Salesse et al. 2021	Use of body<br>Play a mirror game with a confederate. Participant were given the instruction of "imitate each other, create synchronized and interesting motions, and enjoy playing together". The mirror game allows capturing both socio-motor improvisation and socio-motor synchronization. Improvising implies the production of "various, complex and interesting" Socio-Motor Improvisation in Schizophrenia movements but risking being poorly synchronized. At the same time, being synchronous implies the production of simultaneous motion but risking decreasing the improvisation. Solving this trade-off between improvisation and synchronization in the mirror game reflects the core of our experimental protocol.	Improvisation
Sampedro et al. 2019	Multimodal<br>Participants were asked to write all unusual uses for Cardboard Boxes that they could think of.	TTCT + Unusual Uses subtest
Son et al. 2015	Quantity of responses produced for each request	Design Ti+IdeaTi+VerbalC+VerbalL
Wang et al. 2017	Written<br>Total number of responses given at the alternative uses test	Fluency at *Alternative uses test*

Table-Suppl5. Data coding for originality, intended as the statistical rarity of the answer.

Abraham et al. 2007*	Manipulation of figures<br>How unusual and unique the invention is<br>Verbal written<br>The infrequency of given solutions when participants was asked to generate as many uses as possible for three common objects	Score "originality" at the *Creative imagery test*<br>+<br>Score "originality" at the *Alternate uses test*

Del Missier et al. 2022	Verbal written<br>The infrequency of given solutions when participants was asked to generate as many uses as possible for three common objects<br>+<br>Originality ratings of the most original responses	Score "average originality" at the *Alternate uses test*<br>+<br>Score Peak Originality at the *Alternate uses test*
Mavrogiorgou et al. 2021	Statistical rarity of answer	TTCT
Sampedro et al. 2019	1 point for original or uncommon responses, and 0 points for unoriginal responses	TTCT + Unusual Uses subtest
Wang et al. 2017	Statistical rarity of answer	Originality figure completion + Tangram construction + story generation

* According to the Cochrane Handbook for Systematic Reviews of Interventions, when a primary study reports more than one result for the same outcome—such as measurements taken at different time points—it is recommended to either select a single relevant time point or to compute the average of the effect estimates across time points (Higgins et al., version 6.5, 2024).

Borenstein et al. (2021) argue that this situation is conceptually analogous to cases where a primary study provides more than one valid measure of the same outcome (e.g., using different instruments) at a single time point. In both cases, the effect sizes are statistically dependent, as they derive from the same sample of participants. Consequently, Borenstein et al. recommend computing a synthetic (averaged) effect size and its corresponding variance to represent the outcome more accurately in the meta-analysis.

In line with this recommendation, when a primary study in our review reported two statistically dependent effect sizes for the same outcome, and both were deemed valid and relevant, we opted—where possible—to compute an average effect size using the formulas provided in Borenstein et al. (2021). This approach allowed us to retain more information from the primary studies while appropriately accounting for statistical dependence.

§S4. Sensitivity and cumulative analyses

Table-Suppl6. Sensitivity analysis for "creativity" outcome. Details.

**Study**	**k**	**ES**	**LL**	**UL**	**Sig.**	**V**	**SE**	**N**	**n1**	**n2**	**n_na**

Abraham et al. 2007	10	-0,71	-0,98	-0,43	0	0,02	0,14	681	257	424	0
Del Missier et al. 2022	10	-0,7	-0,96	-0,44	0	0,02	0,13	693	268	425	0
Folley and Park 2005	10	-0,83	-1,15	-0,51	0	0,03	0,16	693	268	425	0
Jaracz et al. 2012	10	-0,83	-1,16	-0,51	0	0,03	0,17	639	242	397	0
Keefe and Magaro 1980	10	-0,85	-1,15	-0,55	0	0,02	0,15	697	265	432	0
Mavrogiorgou et al. 2021	10	-0,8	-1,13	-0,48	0	0,03	0,16	692	275	417	0
Michalica and Hunt 2013	10	-0,82	-1,14	-0,5	0	0,03	0,16	584	275	309	0
Rodrigue et al. 2012	10	-0,79	-1,12	-0,46	0	0,03	0,17	675	263	412	0
Salesse et al. 2021	10	-0,82	-1,15	-0,49	0	0,03	0,17	669	255	414	0
Sampedro et al. 2019	10	-0,81	-1,15	-0,48	0	0,03	0,17	637	240	397	0
Wang et al. 2017	10	-0,75	-1,07	-0,43	0	0,03	0,16	610	242	368	0

Table-Suppl7. Cumulative analysis for "creativity".

**Study**	**Mod.**	**ES**	**LL**	**UL**	**Sig.**	**V**	**SE**	**N**	**n1**	**n2**	**n_na**
Keefe and Magaro 1980	23,8	0	-0,75	0,76	0,992	0,15	0,39	30	20	10	0
Wang et al. 2017	30,23	-0,61	-1,73	0,5	0,28	0,32	0,57	147	63	84	0
Salesse et al. 2021	33,8	-0,64	-1,25	-0,02	0,042	0,1	0,31	205	93	112	0
Aracz et al. 2012	36	-0,6	-1,04	-0,16	0,008	0,05	0,23	293	136	157	0

Michalica and Hunt 2013	37,7	-0,59	-0,96	-0,23	0,001	0,03	0,19	436	146	290	0
Folley and Park 2005	39,5	-0,57	-0,89	-0,26	0	0,03	0,16	470	163	307	0
Mavrogiorgou et al. 2021	40,4	-0,59	-0,87	-0,31	0	0,02	0,14	505	173	332	0
Sampedro et al. 2019	40,4	-0,6	-0,83	-0,37	0	0,01	0,12	595	218	377	0
Abraham et al. 2007	43,07	-0,69	-0,97	-0,4	0	0,02	0,15	641	246	395	0
Rodrigue et al. 2012	43,7	-0,7	-0,96	-0,44	0	0,02	0,13	693	268	425	0
Del Missier et al. 2022	51,94	-0,79	-1,09	-0,49	0	0,02	0,15	727	285	442	0

Table-Suppl8. Sensitivity analysis for elaboration. Details of data. Sensitivity analysis confirmed the stability of the negative effect on elaboration scores.

**Study**	**k**	**ES**	**LL**	**UL**	**Sig.**	**V**	**SE**	**N**	**n1**	**n2**	**n_na**
Abraham et al. 2007	4	-0,42	-0,94	0,1	0,11	0,07	0,26	245	107	138	0
Folley and Park 2005	4	-0,61	-0,96	-0,27	0,001	0,03	0,18	257	118	139	0
Mavrogiorgou et al.	4	-0,46	-0,89	-0,03	0,037	0,05	0,22	287	133	154	0
Sampedro et al. 2019	4	-0,54	-1,06	-0,02	0,041	0,07	0,26	201	90	111	0
Wang et al. 2017	4	-0,3	-0,61	0,02	0,063	0,03	0,16	174	92	82	0

Table-Suppl9. Sensitivity analysis for “flexibility”.

**Study**	**K**	**ES**	**LL**	**UL**	**p**	**Var**	**SE**	**N**	**n1**	**n2**	**n_na**
**Del Missier et al. 2022**	6	-0.41	-0.64	-0.19	0.0	0.01	0.12	488	220	268	0
**Folley and Park 2005**	6	-0.44	-0.7	-0.18	1	0.02	0.13	488	220	268	0
**Jaracz et al. 2012**	6	-0.55	-0.86	-0.24	0.0	0.02	0.16	434	194	240	0
**Kucway et al. 2023**	6	-0.58	-0.84	-0.32	0.0	0.02	0.13	398	175	223	0
**Mavrogiorgou et al. 2021**	6	-0.53	-0.82	-0.24	0.0	0.02	0.15	487	227	260	0
**Sampedro et al. 2019**	6	-0.46	-0.76	-0.17	2	0.02	0.15	432	192	240	0
**Wang et al. 2017**	6	-0.52	-0.85	-0.2	2	0.03	0.17	405	194	211	0

Table-Suppl10. Sensitivity analysis for “fluency”.

**Study**	**k**	**ES**	**LL**	**UL**	**Sig.**	**V**	**SE**	**N**	**n1**	**n2**	**n_na**
**Abraham et al. 2007**	7	-0.71	-1.1	-0.31	0.0	0.04	0.2	473	221	252	0
**Del Missier et al. 2022**	7	-0.69	-1.05	-0.32	0.0	0.03	0.19	485	232	253	0
**Jaracz et al. 2012**	7	-0.94	-1.36	-0.51	0.0	0.05	0.22	431	206	225	0

**Keefe and Magaro 1980**	7	-0.92	-1.35	-0.49	0.0	0.05	0.22	489	229	260	0
**Mavrogiorgou et al. 2021**	7	-0.9	-1.35	-0.46	0.0	0.05	0.23	484	239	245	0
**Sampedro et al. 2019**	7	-0.83	-1.33	-0.34	1	0.06	0.25	429	204	225	0
**Son et al. 2015**	7	-0.81	-1.29	-0.33	1	0.06	0.24	440	206	234	0
**Wang et al. 2017**	7	-0.87	-1.37	-0.36	1	0.07	0.26	402	206	196	0

§S5. Moderator analyses

Table-Suppl11. Moderator analyses. Among the variables tested, only the mean age of the experimental group emerged as a statistically significant moderator.

		**Creativity**	**Elaboration**	**Flexibility**	**Fluency**	**Originality**
**Publication year**	**k**	11	5	7	8	5
	**Int.+Slope**	Y= -39+0.02X	Y= 52+ -0.03X	Y= -68.59+0.03X	Y= -143.24+0.07X	Y= -29.39+0.01X
	**Sig.**	0.508	0.479	0.213	0.131	0.644
**Age of patients**	**k**	11	5	7	8	5
	**Int.+slope**	Y= 1.12+ -0.05X	Y= -2.06+0.04X	Y=0.98+ -0.04X	Y= 1.75+ -0.07X	Y= -0.28+ -0.01X
	**Sig.**	0.04	0.331	0.035	0.026	0.663
**Mean difference in age between patients and control group**	**k**	11	5	7	8	5
	**Int.+Slope**	Y= -0.74+ -0.01X	Y= -0.25+-0.05X	Y= -0.46+-0.02X	Y= -0.78+ -0.04X	Y= -0.55+ -0.02X
	**Sig.**	0.616	0.406	0.587	0.368	0.532
**Mean age in healthy group**	**k**	11	5	7	8	5
	**Int.+Slope**	Y= -0.23+ -0.02X	Y= -1.31+0.03X	Y= 0.13+ -0.02X	Y= 0.34+ -0.03X	Y= -0.62+ 0.00X
	**Sig.**	0.392	0.335	0.240	0.298	0.998
**Male proportion in the experimental group**	**k**	8	3	5	5	3
	**Int.+Slope**	Y= -0.95+0.05X	Y= -1.35+1.31X	Y= -0.07+ -0.74X	Y= 1.2+ -3.95X	Y= -0.47+ -0.52X
	**Sig.**	0.977	0.671	0.737	0.145	0.614
**Years of education of patients**	**k**	7	3	5	4	No data available
	**Int.+Slope**	Y= 0.67+ -0.10X	Y= 1.29+ -0.13X	Y=0.17+ -0.05X	Y= -0.39+ -0.01X	No data available
	**Sig.**	0.256	0.626	0.421	0.964	No data available

**Mean difference in years of education between patients and healthy controls**	**k**	6	3	5	4	No data available
	**Int.+Slope**	Y= -0.67+ -0.05X	Y= -0.54+0.10X	Y= -0.54+ -0.04X	Y= -0.45+0.03X	No data available
	**Sig.**	0.582	0.679	0.627	0.837	No data available

Supplementary Figures – Captions

Figure-Suppl1. Sensitivity analysis for "creativity" outcome. Plot.

Figure-Suppl2. Publication year. A weighted meta-regression using publication year as a predictor (random-effects model) showed no significant trend over time.

Figure-Suppl3. Sensitivity analysis for "elaboration".

Figure-Suppl4. Cumulative analysis for "elaboration".

Figure-Suppl5. Sensitivity analysis for "flexibility". Plot.

Figure-Suppl6. Sensitivity analysis for "fluency".
