# Supplementary figures and images for: A meta-analysis and systematic review of creativity in schizophrenia: toward an ecological understanding integrating clinical and philosophical perspectives
Source: Front Psychol. 2026 Mar 4;17:1658295. doi: 10.3389/fpsyg.2026.1658295 (PMC12997125; doi:10.3389/fpsyg.2026.1658295)

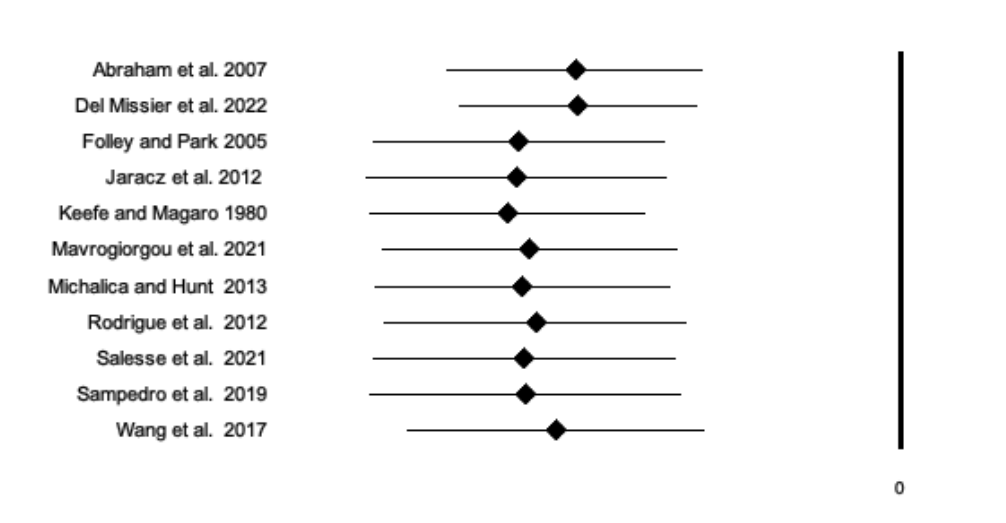

Supplement: Supplementary file 2 [file Image_1.tiff]

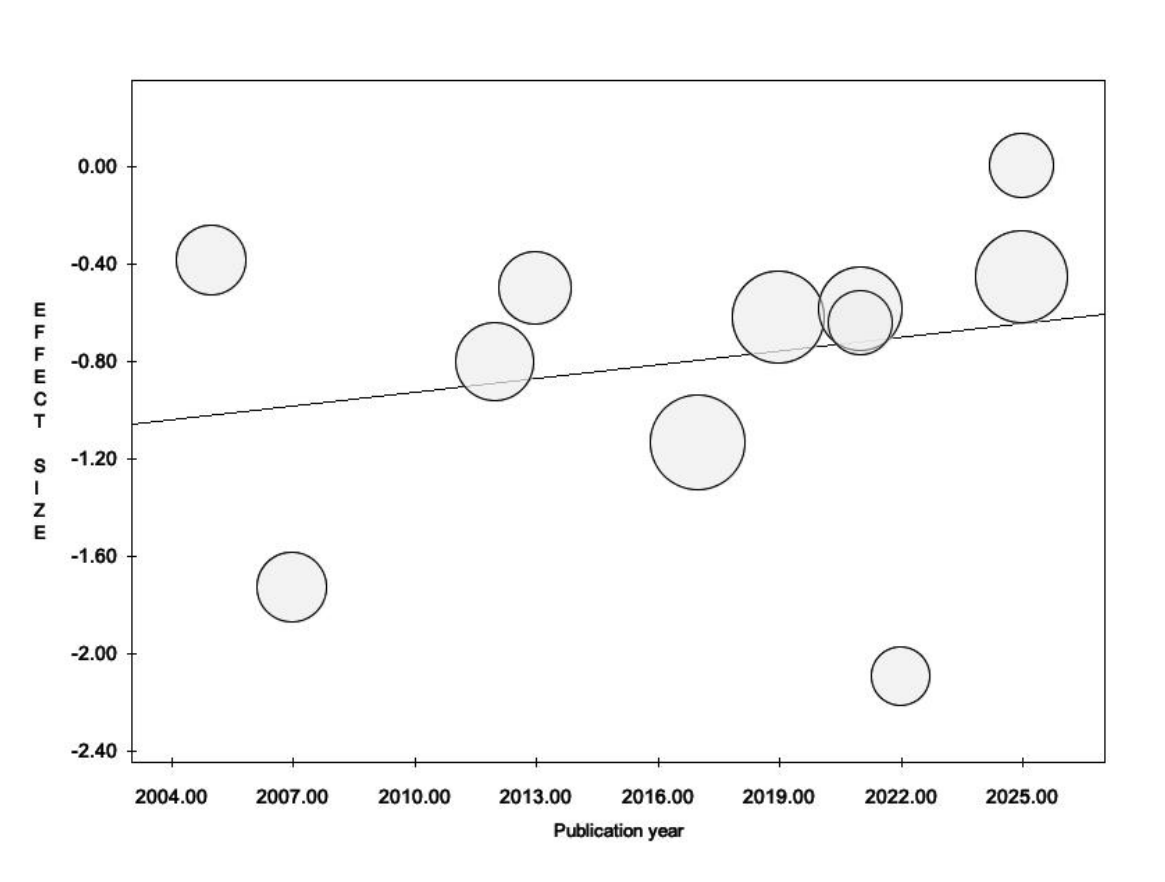

Supplement: Supplementary file 3 [file Image_2.tiff]

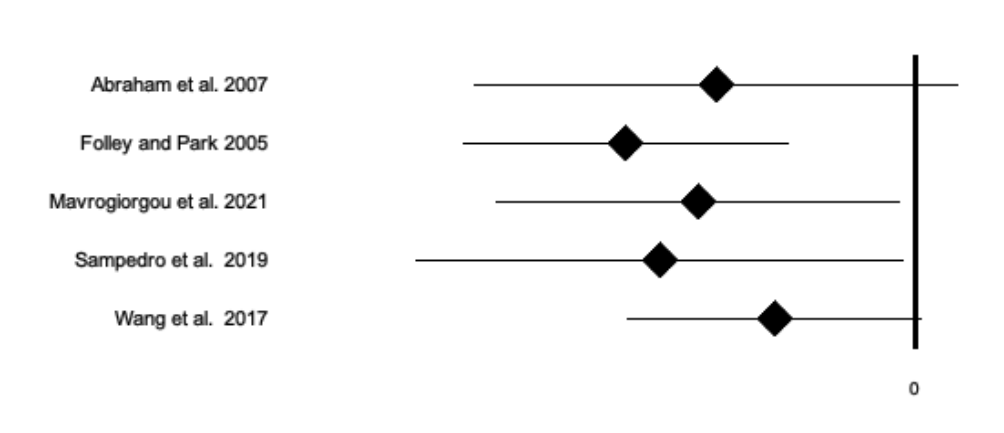

Supplement: Supplementary file 4 [file Image_3.tiff]

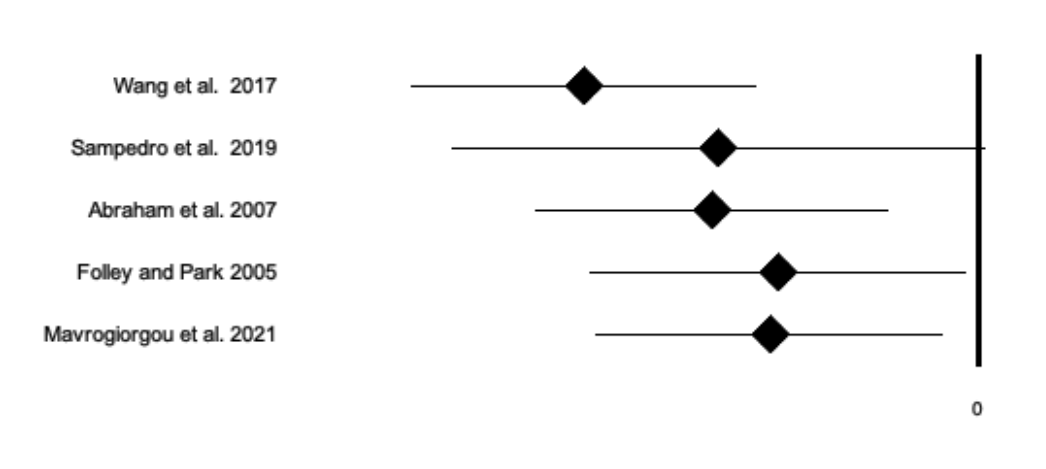

Supplement: Supplementary file 5 [file Image_4.tiff]

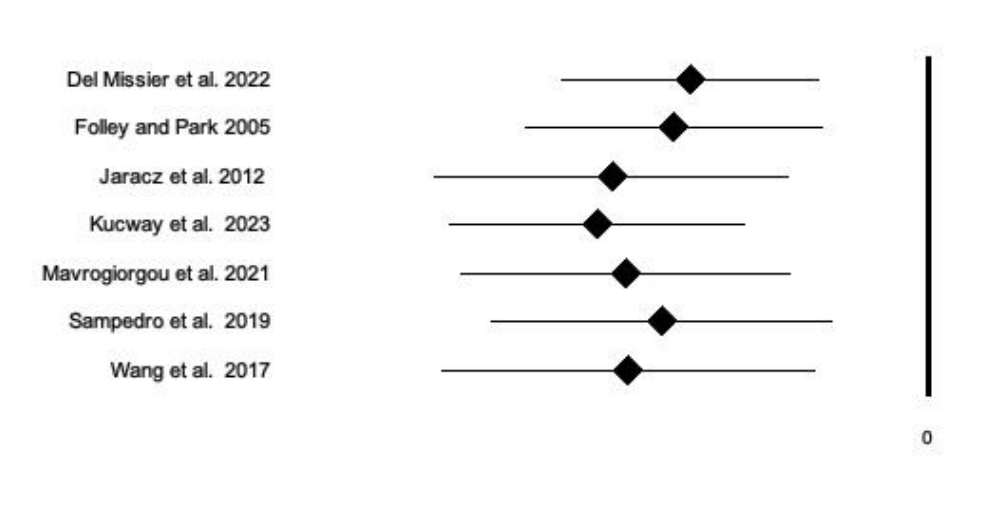

Supplement: Supplementary file 6 [file Image_5.tiff]

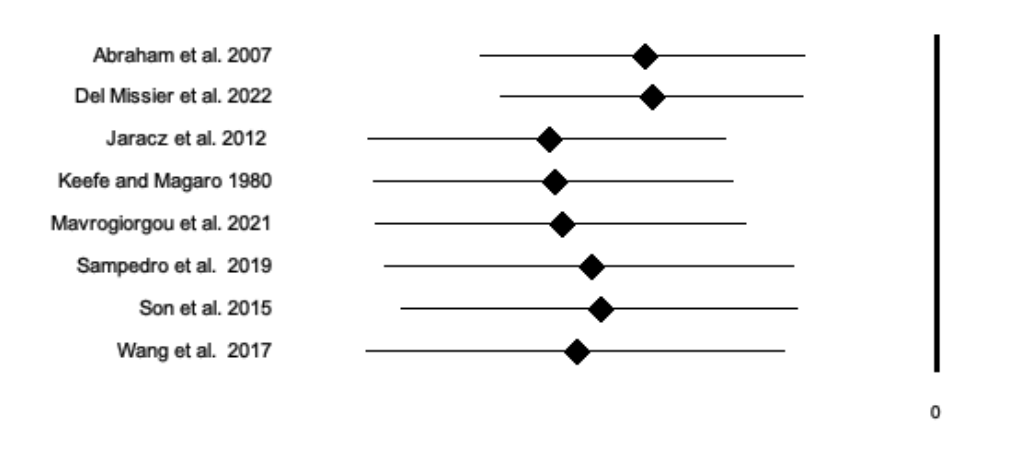

Supplement: Supplementary file 7 [file Image_6.tiff]
